# Targeting Protein-Protein Interactions for Parasite Control

**DOI:** 10.1371/journal.pone.0018381

**Published:** 2011-04-27

**Authors:** Christina M. Taylor, Kerstin Fischer, Sahar Abubucker, Zhengyuan Wang, John Martin, Daojun Jiang, Marc Magliano, Marie-Noëlle Rosso, Ben-Wen Li, Peter U. Fischer, Makedonka Mitreva

**Affiliations:** 1 Department of Genetics, The Genome Center, Washington University School of Medicine, St. Louis, Missouri, United States of America; 2 Infectious Diseases Division, Department of Internal Medicine, Washington University School of Medicine, St. Louis, Missouri, United States of America; 3 INRA 1301, CNRS 6243, UNSA, Interactions Biotiques et Santé Végétale, Sophia-Antipolis, France; Naval Research Laboratory, United States of America

## Abstract

Finding new drug targets for pathogenic infections would be of great utility for humanity, as there is a large need to develop new drugs to fight infections due to the developing resistance and side effects of current treatments. Current drug targets for pathogen infections involve only a single protein. However, proteins rarely act in isolation, and the majority of biological processes occur via interactions with other proteins, so protein-protein interactions (PPIs) offer a realm of unexplored potential drug targets and are thought to be the next-generation of drug targets. Parasitic worms were chosen for this study because they have deleterious effects on human health, livestock, and plants, costing society billions of dollars annually and many sequenced genomes are available. In this study, we present a computational approach that utilizes whole genomes of 6 parasitic and 1 free-living worm species and 2 hosts. The species were placed in orthologous groups, then binned in species-specific ortholgous groups. Proteins that are essential and conserved among species that span a phyla are of greatest value, as they provide foundations for developing broad-control strategies. Two PPI databases were used to find PPIs within the species specific bins. PPIs with unique helminth proteins and helminth proteins with unique features relative to the host, such as indels, were prioritized as drug targets. The PPIs were scored based on RNAi phenotype and homology to the PDB (Protein DataBank). EST data for the various life stages, GO annotation, and druggability were also taken into consideration. Several PPIs emerged from this study as potential drug targets. A few interactions were supported by co-localization of expression in *M. incognita* (plant parasite) and *B. malayi* (*H. sapiens* parasite), which have extremely different modes of parasitism. As more genomes of pathogens are sequenced and PPI databases expanded, this methodology will become increasingly applicable.

## Introduction

Roundworm and flatworm infections, known as helminth infections, are an enormous problem worldwide, especially in developing countries. About one-third of earth's population are infected with parasitic helminths[1]. These parasite infections can range from diseases such as elephantiasis and river blindness[2] to detrimental effects on child development and health[3]. Further, helminths have devastating effects on crops, costing $78 billion per year globally[4], and infect domesticated animals, which costs billions of dollars[5]. Anthelminthic drug resistance is an increasing problem[6], so pesticide, drug and vaccine development for parasite infections would have a great impact on improving world health and productivity.

With recent whole genome sequencing efforts, several parasitic genomes have been sequenced and much information important for drug discovery can be mined[7]. Several published reports used genomic data to prioritize parasitic drug targets using three main approaches. One method examined all genes in the genome encoding specific types of receptors known to be important for parasitic survival[8], [9]. Metabolic chokepoints or essential proteins in metabolic pathways have been targeted for drug prioritization[10], [11], [12], [13]. A third approach determined orthologous groups of proteins in various parasites, model organisms, and humans. The orthologous protein groups were used to extract experimental information, such as RNAi and expression data, for prioritization or to place higher priority on proteins that were not in the host genomes or have high homology to the PDB (Protein DataBank) [14], [15], [16], [17], [18].

These previous drug prioritization approaches target single specific proteins. In fact, the traditional approach for drug discovery involves targeting a single enzyme active site with a small molecule[19]. However, proteins rarely act in isolation and often interact with other proteins to accomplish their biological function, forming protein-protein interaction (PPI) networks[20]. Given large-scale genomics and proteomics initiatives, entire interactomes have been identified, leading to important insights into biological pathways and host-pathogen interaction[21]. PPIs are of central importance and are involved in nearly all cellular processes[22], making these interactions important targets for drug discovery[23]. While PPIs are challenging targets due to the large surface area and shallow interaction at the protein-protein interface, their recent success as drug targets has been reported[19]. Targeting PPIs targets can increase the number of drug targets dramatically[24] and offer the next large innovation in drugs that will be released in the next decade[21].

The increase in the number of full genome sequences and various PPI databases presents an opportunity to apply a computational approach to find novel PPI drug targets. By using all complete genomes of roundworms (Nematoda) and flatworms (Platyhelminthes) available presently, we have identified PPIs unique to certain groups of parasites or that have molecular features unique to Nematoda and/or Platyhelminthes relative to their hosts. Several PPIs were tested via *in situ* hybridization to confirm the co-localization of protein expression in the human parasite *Brugia malayi* and the plant parasite *Meloidogyne incognita*. The methodology used for drug prioritization in this study was guided by the wealth of functional information available for the model nematode *Caenorhabditis elegans.* While as a prototype we used species from two phyla, the approach could be applied to any phyla in which several pathogenic species have a sequenced genome.

## Results

### Orthologous groups

**Figure 1:**
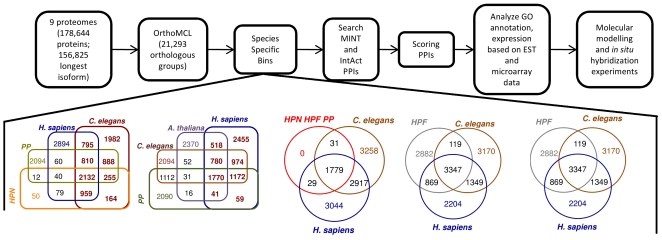
Flow chart of methodology to find PPI drug targets. The longest protein isoform in nine different proteomes were placed in orthologous groups. The orthologous groups were placed in species-specific bins (The numbers of orthologous groups within species-specific bins are shown in the Venn diagrams below). Protein interaction data from MINT and IntAct were used to find groups that contained PPIs. The PPIs were scored and analyzed based on GO annotation, ESTs, and microarray data, then characterized using molecular modeling and experimental techniques.

**Figure 2:**
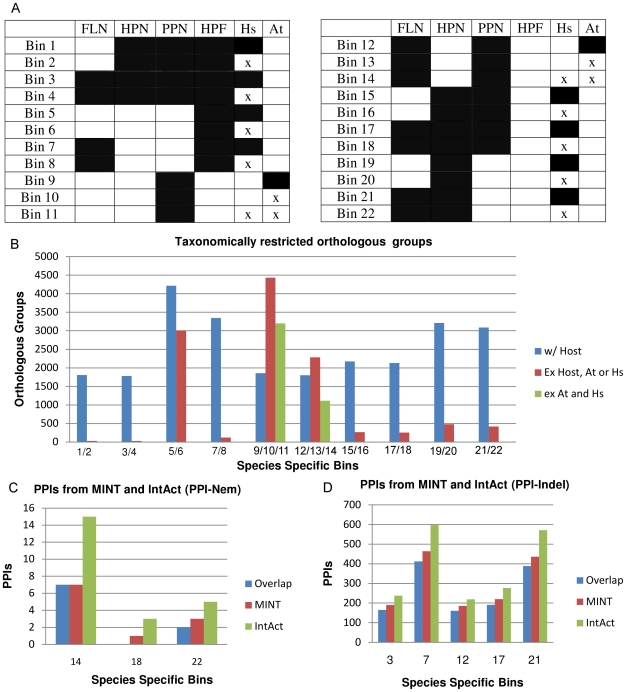
Results of orthologous groups. A. Taxonomically restricted orthologous groups, based on OrthoMCL output, were parsed for PPIs. The following abbreviations were used: HPN (human parasitic nematode): *B. malayi* and *T. spiralis*, FLN (free-living nematode): *C. elegans*, PPN (plant parasitic nematode): *M. incognita* and *M. hapla*, HPF (human parasitic flatworms): *S. mansoni* and *S. japonicum*, Hs: *H. sapiens*, At: *A. thaliana*, B. Distribution of orthologous groups within taxonomically restricted orthologous bins based on whether the host is included or excluded, C. Number of MINT and IntAct PPIs in species-specific binds from the PPI-Nem group, D. Number of MINT and IntAct PPIs in species-specific bins from the PPI-Indel group.

Markov Clustering of the 156,825 proteins (originating from 9 species (Table 1)) resulted in 21,293 orthologous groups (available on nematode.net[25]) which were placed in species specific bins and enumerated (Figure 1 & 2A,B). The number of orthologous groups in the bins decreases dramatically, in some cases more than 10 fold, when helminth-specific proteins are allowed in a bin (i.e. host proteins are excluded). The number of orthologous groups decreases less than 21% when a *C. elegans* protein is required in the orthologous group. However, the number of orthologous groups with platyhelminths significantly decreases when *C. elegans* are included and humans excluded. The large number of orthologous groups that contain a host ortholog in addition to the set of nematode and platyhelminth proteins shows that when proteins are conserved across a phyla, they are likely to be conserved in other branches of the phylogenic tree. Relatively few orthologous groups specific to both nematodes and platyhelminthes lack an ortholog in the human host. These proteins are of interest, as they are taxonomically restricted and conserved among helminths, and therefore, they are possible targets for broad and general control of helminths. Interestingly, there are more orthologous groups when *Arabidopsis thaliana* is excluded from the orthologous group, compared to being included, with the plant parasites compared, indicating more unique nematode–specific proteins when compared to plant. This is not surprising because *A. thaliana* (kingdom Viridiplantae) is much more evolutionarily divergent than humans and worms (kingdom Metazoa). Hence, based on our results, targeting the plant parasite-specific proteins has potential to be a fruitful area of research.

**Table 1:** Information regarding parasite and host species studied.

Species	Number of proteins	Number of proteins without isoforms	Trophic ecology^a^	Proteome Resource
***B. malayi***	11,610	11,407	HPN	WS175; www.wormbase.org
***C. elegans***	24,052	20,173	FLN	WS204; www.wormbase.org
***M. hapla***	14,421	14,421*	PPN	http://supfam.mrc-lmb.cam.ac.uk/SUPERFAMILY/cgi-bin/gen_list.cgi?genome=wm
***M. incognita***	20,359	19,212	PPN	http://www.inra.fr/meloidogyne_incognita/genomic_resources/downloads
***S. japonicum***	13,469	13,469*	HPF	v4.0 (http://www.chgc.sh.cn/japonicum/Resources.html)
***S. mansoni***	11,789	11,789*	HPF	v4.0 (http://www.genedb.org/)
***T. spiralis***	16,124	16,124*	HPN	GenBank Acc: ABIR00000000.2^b^
***H. sapiens***	37,868	24,013	Host	(ftp://ftp.ncbi.nih.gov/refseq/H_sapiens/mRNA_Prot/human.rna.gbff.gz)
***A. thaliana***	28,952	26,217	Host	ftp://ftp.ncbi.nih.gov/genomes/Arabidopsis_thaliana/GNOMON and ftp://ftp.arabidopsis.org/home/tair/Sequences/blast_datasets

*Alternative splicing not known, so entire proteome was used.

a HPN – human parasitic nematode, FLN – free-living nematode, PPN – plant parasitic nematode, HPF – human parasitic flatworm.

b The protein set used in this study is slightly larger than what was submitted to GenBank.

### PPIs

PPI databases are heavily populated with *C. elegans* PPIs and have nearly no PPIs for other nematodes and platyhelminths. Therefore, only bins containing *C. elegans* orthologs were analyzed for prioritizing PPIs, (Figure 2C,D). Using multiple databases expanded the number of potential targets due to the unique PPIs per database. Bins where human orthologs were excluded had a smaller number of PPIs than bins where proteins with human orthologs were considered. The bins where human orthologs were included had many more PPIs, yielding more potential drug targets. In the PPI databases, a small number of PPIs were mapped versus the total number of proteins in the proteome (*C. elegans* has 24,052 proteins of which 4,159 are in the IntAct PPIs and 3,257 in the MINT PPIs), but the numbers are expected to increase as more experimental PPIs are discovered. Based on our scoring function (*see* Methods) we provide a ranked list of potential PPI drug targets (Table 2). The results of Bin 12 for PPI-Indel1 and PPI-Indel2 (*see* Methods) are also presented in a ranked list in the Tables S1, S2, and S3. Table S4 provides the score broken into various terms to aid in evaluating each PPI.

**Table 2:** The top 8 PPI targets in each of the three major groups: specific to nematodes (PPI-Nem), where both proteins contain indels with respect to human host (PPI-Indel2), with one indel with respect to human host (PPI-Indel1).

PPI	Score	RNAi Pheno^a^	PDB Homo.	Frac. of Len	PPI Group^b^	Function	Stage^c^Localization^d^
**Q03601/** **Q20329**	**253.7**	**21/** **315**	**31.3/** **30**	**0.81/** **0.65**	**Nem** **I**	**ZnFinger, NHL repeat/** **actin-like**	**L1,Em,A / L1,L4,Eg,Em,A** **PMR / ---**
O45666*/O45666*	237.8	32156/32156	35/35	0.53/0.53	NemIM	NHR-Znfinger/NHR-Znfinger	L1,L2,L4,Em,A / L1,L2,L4,Em,A--- / ---
O45666*/Q09528*	156.9	32156/No	35/25.9	0.53/0.50	NemIM	NHR-Znfinger/NHR-Znfinger	L1,L2,L4,Em,A / L,A--- / ---
Q21234*/Q21234*	150.0	215/215	No	No	NemIM	Integrase	L1,L2,L3,L4,Em,A/L1,L2,L3,L4,Em,A--- / ---
Q8MYQ1/Q22631	147.4	7/No	39.6/30.1	0.96/0.54	NemIM	Ser-kinase/thrombospondin	L1 / L1,L2,L4,Em--- / ---
O01489/O01489	135.0	2/2	No	No	NemIM	ZnF protein	L1,L2,L3,L4,Em,A / L1,L2,L3,L4,Em,APIMRNH / PIMRNH
Q03601/O16266	75.0	21/No	No	No	NemI	ZnFinger, NHL repeat/	L1,Em,A / EmPMR / ---
Q9NDH1/Q93413	67.5	No/254	No	No	NemM	RNA-dep-RNA-pol/DNA-RNA helicase	L1,L2,L3,L4,Em,A / L1,L2,L3,L4,Em,A--- / S
Q93716^+^/Q93716^+^	449.6	321576/321576	100/100	0.99/0.99	Indel2IM	NUDIX hydrolase domain	L1,Em,A / L1,Em,APMH / PMH
P91988^+^/P91988^+^	449.5	315/315	100/100	0.99/0.99	Indel2I	Flavoprotein	L1,Em,A / L1,Em,A--- / ---
Q20471^+^/Q20471^+^	447.3	321546/321546	100/100	0.97/0.97	Indel2IM	Protein kinase	L1,L4,Em,A / L1,L4,Em,A--- / ---
P91851^+^/P91851^+^	434.6	2/2	100/100	0.99/0.99	Indel2I	nicotinate-nucleotide adenylyltransferase	Em,A / Em,A--- / ---
O18209^+^ */Q17796^+^	407.3	321/321546	28/41.5	0.51/0.95	Indel2IM	Protein Kinase/Zinc finger	L1,L4,Em,A / L1,L4,Em,AE / EHIPR
P46822^+^/P46822^+^	396.3	2/2	78.6/78.6	0.83/0.83	Indel2IM	Tetratricopeptide	L1,L4,Eg,Em,A / L1,L4,Eg,Em,A--- / ---
**P46822^+^/** **Q17581^+^**	**392.7**	**2/** **32154**	**78.6/** **50.8**	**0.83/** **0.88**	**Indel2** **IM**	**Tetratricopeptide/** **Bromodomain**	**L1,L4,Eg,Em,A/L1,Em,A** **--- / ---**
O62305^+^ */O62305^+^ *	391.4	No/No	100/100	0.91/0.91	Indel2I	Protein kinase	L1,L4,Em,A / L1,L4,Em,A--- / ---
P34475^+^ */Q19207*	475.9	3217/321546	99.5/99.7	0.99/0.53	Indel1IM	Tubulin/ Hydroxymethylglutaryl-CoA reductase	L1,L4,Em,A / L1,L2,Eg,Em,ARE / ---
***O01427^+^*** * ***/*** ***Q19126***	***434.5***	***32174/*** ***3215***	***100/*** ***86.4***	***0.99/*** ***0.83***	***Indel1*** ***IM***	***Protein kinase/*** ***ATPase***	***L1,L4,Em,A / L1,L4,Eg,Em,A*** ***RE / whole body***
P39745/Q9BIB3^+^ *	426.9	32157/3215	100/100	0.99/0.54	Indel1IM	Protein kinase/Lipase	L1,L2,L4,Em,A / L1,A--- / ---
**Q95005/** **Q19207^+^** *	**426.0**	**32156/** **321546**	**100/** **99.7**	**0.99/** **0.53**	**Indel1** **IM**	**Proteasome/Hydroxymethylglutaryl-CoA reductase**	**L1,L4,Em,A / L1,L2,Eg,Em,A** **PM / ---**
Q19207^+^ */Q22799	425.7	3215746/321546	99.7/100	0.53/0.98	Indel1IM	Hydroxymethylglutaryl-CoA reductase/Dynein light chain	L1,L2,Eg,Em,A / A--- / PNI
P39745*/O62305^+^ *	420.6	32157/No	100/100	0.99/0.91	Indel1IM	Protein kinase like /Protein kinase	L1,L2,L4,Em,A / L1,L4,Em,A--- / ---
P39745*/O16299^+^	413.6	32157/3	100/65	0.99/0.98	Indel1IM	Protein kinase – like /MCM protein 7	L1,L4,Em,A / L1,L2,L4,Em,A--- / ---
P34442^+^ */Q27488	406.1	21/32	40.3/100	0.88/0.99	Indel1IM	Protein-tyrosine phosphatase / Proteasome	L1,L4,Em,A / L1,L4,EgEm,AEP / ---

The full list is in Table S1.

*indicates druggable, PPIs in bold italic were tested with FISH, and + indicates protein with indel, ^a^ RNAi phenotype 1 = Larval/Adult Lethal/Arrest, 2 = Embryonic Lethal, 3 = Sterility, 4 = Morphology, 5 = Growth, 6 = Movement, 7 = Vulva, 8 = Other; ^b^ Indicates analysis group (Nem, Indel2, and Indel1) and also the database where the PPI was found (M = MINT and I = IntAct), ^c^ Stages are listed as L1, L2, L3, L4, egg (Eg), embryo (Em), and Adult (A), ^d^ Localization in *C. elegans* listed as pharynx (P), intestine (I), reproductive (R), muscle (M), hypodermis (H), nervous system (N), somatic (S), embryo (E).

### Potential Drug Targets

#### PPI-Indel1

PPIs in PPI-Indel1 provide a unique position to specifically target the interaction with helminth proteins without disrupting the interaction between orthologous human proteins. The interaction between O01427 and Q19126 was found in *C. elegans* via yeast two hybrid[26] and was chosen for FISH testing in *B. malayi* (Table 2) because of the *C. elegans* expression data available. Found in Bins 3, 7, 17, 21, both proteins in this interaction have very severe RNAi phenotypes in *C. elegans* and high homology to the PDB. O01427 has more interactions (5) than Q19126 (1). Both transcripts were found to be differentially expressed in the large roundworm, *Ascaris suum,* male intestines and head. In *C. elegans*, O01427 is localized to meiotic chromosomes in both the oocytes and gonads; Q19126 was expressed widely and continuously in the entire body in *C. elegans*. O01427 functions as a serine/threonine kinase (IPR008271, IPR017442, IPR002290), which plays an important role activating several downstream pathways. Q19126 is a protein associated with ATPase synthase (IPR008688) and is part of subunit b of the peripheral stalk on F-ATPase. O01427 is considered a druggable domain by Hopkins criteria and has two deletions relative to vertebrate homologs which were modeled for further characterization.

**Figure 3:**
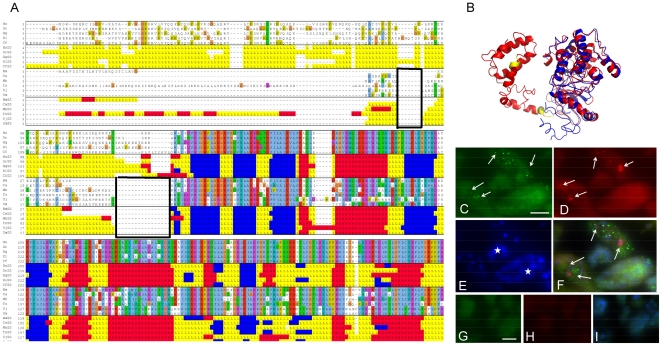
Characterization of O01427/Q19126. **A.** Multiple sequence and secondary structure alignment of vertebrate reference sequences with selected nematode sequences. Within the secondary structure alignments, the random coiled regions are shown in yellow, the beta sheets are shown in blue and helices are shown in red. The two boxed regions show the deletions in the worms relative to the vertebrates. **B.** Predicted 3D structure of O01427 (*H. sapiens* protein in the orthologous group (red), *B. malayi* protein (blue), and indels (yellow)). **C**. Granular staining (arrows) for Q19126 [XP_00189449.1] mRNA in the cytoplasm of morula stage embryos in the midbody region of a female *B. malayi*. The biotin labeled probe was detected using AlexaFluor 488-labeled streptavidin (green). **D.** Identical section as in **C** showing granular staining (arrows) for O01427 [XP_001892118.1] mRNA in the same embryos. The digoxygenin labeled probe was detected using a Rhodamin conjugated anti-digoxygenin antibody (red). **e.** Identical section as in **c** but DAPI stain (blue) showing differential degrees of chromatin condensation in the embryos. **f.** Overlay of **c-e** showing co-localization of mRNA expression of Q19126 and O01427 (arrows) especially in embryos with less densely condensed chromatin. Hybridization of sense probe for Q19126 (**g.**) and O01427 (**h.**) on a serial section to **c** showing the absence of a specific labeling. **i.** Overlay of **g** and **h** including a DAPI stain (blue) showing the morula stage embryos with different degrees of chromatin condensation but the absence of a specific hybridization signal. Scale bar 10 µm.

TASSER-Lite was used to create the homology model because an *ab initio* approach was needed to model unresolved structure of the variable-length N-terminal end where the indels were located. The longest *H. sapiens* sequence (host) in the orthologous group and *B. malayi* (parasite and subject of FISH experiment) were modeled. The *H. sapiens* predicted structure has two small indels that are not found in nematodes (Figure 3A,B and Figure S1). The secondary structure prediction of the N-terminal end is mainly a loop with no defined beta sheet or alpha helical structure and was unresolved in the X-ray crystal structure, which may indicate disorder; the secondary structure of the remainder of the protein is conserved. However, two independent predictors suggested the *H. sapiens* protein has mainly alpha-helical character. Like many PPIs, this protein might undergo a disorder-to-order transition upon binding its protein partner[27], making the PPI target easy to disrupt. The difference in the disordered region and indels between *H. sapiens* and nematodes provides a specific mechanism for targeting O01427/Q19126.

The *C. elegans* interaction between O01427 and Q19126 was tested in *B. malayi* using FISH. Both FISH antisense probes showed a distinct and tightly developmentally regulated mRNA hybridization pattern (Fig. 3C, Figure S2 and Video S1). Expression of the Q19126[XP_00189449.1] gene was mainly detected in egg cells and developing embryos. Weak staining was observed in the hypodermal region and the uterus epithelium (Figure S2). Although multiple sections of female and male *B. malayi* worms were examined for the expression of the Q19126[XP_00189449.1] gene, the most striking signal was obtained in the uterine egg cells and developing embryos. Therefore, the further analysis was focused on these structures. Expression of the O01427[XP_001892118.1] gene was slightly weaker for the Q19126[XP_00189449.1] gene and almost exclusively confined to egg cells and embryos. However, in the morula stage, embryo expression of both genes was clearly co-localized, especially in areas with less densely condensated chromatin (Figure 3C, Figure S2 F,G). The intracellular distribution of both messages was distinct within the cytoplasm. Localization of gene expression in the same tissue within *B. malayi*, in addition to the PPI between O01427 and Q19126 found in *C. elegans* via yeast 2-hybrid, we surmise that this PPI likely exists in other nematode species and may be a good drug target.

#### PPI-Indel2

The interaction between P46822 (insertion) and Q17581 (deletion) had the most promising expression data from PPI-Indel2 over many life stages in *B. malayi* (Table 2). As a kinesin light chain, P46822 makes many PPIs in the cell to carry out its function and this is reflected in the number of PPIs emanating from this hub with 20 and 21 PPIs in MINT and IntAct, respectively. Typically moving from the minus end to the plus end of a microtubule, the kinesin light chain binds to the cargo being transported. Q17581 makes 2 PPIs in MINT and IntAct. The NCBI KOG (clusters of euKaryotic Orthologous Groups) classifies this protein as a CELTIX-1 protein containing a bromodomain that binds to IRF-2, a transcription factor. The bromodomain specifically recognizes acetylated lysines. In addition, co-localization between P46822 and Q17581 was detected by FISH, and a complementary *in situ* method, in pretzel stage embryos of *B. malayi* (Figure S2). While weak labeling of RNA granules was observed in tissues of adult worms, specific expression was found in developing embryos. This target is discussed further in the Text S1. The localization of Q19207/Q95005, another pair within PPI-Indel1, was also found in the same tissue (data not shown).

#### PPI-Nem

**Figure 4:**
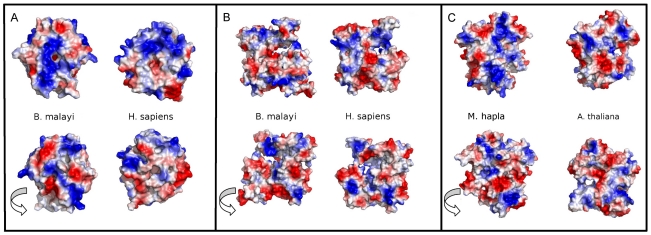
3D homology models of the nematode proteins and the host orthologs for the PPI partners Q03601/Q20329. **A.** *B. malayi* and *H. sapiens* homology models of Q03601; **B.** *B. malayi* and *H. sapiens* homology models of Q20329; and **C.** *M. hapla* and *A. thaliana* homology models of Q20329. All models are colored by electrostatic potential in vacuum. Q03601 did not have any protein sequences in *A. thaliana* with homology to *M. hapla* and *M. incognita*. Although regions of these proteins have homology to *H. sapiens* (**A** and **B**) and *A. thaliana* (**C**), the charges on the surface of *H. sapiens* and *A. thaliana* proteins are different from the charges on the surface of the nematode proteins. Further, Markov clustering did not group the *H. sapiens* or *A. thaliana* proteins in the same orthologous groups as the nematode proteins.

The majority of hits from PPI-Nem (nematode-specific PPIs) had a uniprot classification of zinc finger, integrase, or serine-kinase (Table 2). Q20329 is an actin protein, and Q03601 has a zinc finger protein NHL-1 at the C-terminal region that forms a beta-propeller twist and contains a coiled-coil region near the N-terminal end of some of the nematode proteins. Co-localization of Q20329/Q03601 was detected in the anterior intestines and pharynx of *M. incognita* (Figure S3, Table S5). Despite both proteins being grouped in a nematode specific bin, the primary sequence similarity results revealed some homology to human proteins. Both proteins were modeled to determine if these proteins differed from the *H. sapiens* proteins enough to be viable drug targets (Figure S4). Differences between the electrostatics on the surface of the nematode proteins themselves and *H. sapiens* reflect opportunities for specific species targeting (Figures 4, S4, S5). Additional information is in the Text S1 and Tables S6, S7, S8, S9, S10, S11.

## Discussion

We demonstrated the validity of a novel approach for genomic-scale prioritization of drug targets applicable for any pathogens of medical and socio-economic importance. The methodology developed takes advantage of the fact that a vast majority of the biological processes occur via interactions of multiple proteins, and therefore the interactions can be considered as putative good targets for developing control strategies. Targeting interactions between proteins with drugs expands the number of drug targets dramatically from which pioneering therapies for pathogens that plague over one-third of the earth's population could be realized. Given the increase in genomic sequencing of nematodes and platyhelminthes, we used comparative genomics to derive novel PPI drug targets from which broad-control strategies could be developed. The methods used in this study are directly pertinent to other parasitic phyla where genomes of several species have been sequenced and will grow in applicability as more genomes and large-scale genomics and proteomics efforts are realized.

There are two major classes of PPIs found in this study: nematode specific proteins (PPI-Nem) and proteins with human orthologs containing nematode specific indels (PPI-Indel). PPI-Nem can be easily targeted without concern because the proteins are specific to nematodes. The PPI-Indel group is quite large and many potential PPIs would be eliminated if these were not considered. Insertions and deletions (indels) are one distinguishing feature that can provide a unique mechanism for targeting proteins in nematodes and platyhelminthes relative to their human orthologs. Indels have been shown to remodel PPI interaction networks[28], and despite the proteins having human orthologs, the proteins may not interact in humans due to the nematode specific indels. Further, there has been some success in targeting the indel region of a protein for drug discovery[29]. Other studies have found that indels were more likely to occur in essential proteins and those that are highly connected[30]. We identified PPI targets that are in both the PPI-Nem and PPI-Indel groups for further testing as drug targets.

Because there is no direct evidence that disrupting the PPIs in this study would have a negative impact on nematode or platyhelminth survival, the assumption was made that if two proteins interacted and had a severe RNAi phenotype in *C. elegans*, the PPI between these two proteins may be important for survival, as well. Further evaluation of the essentiality of the PPI will be necessary when the targets are pursued further. For PPI-Nem, many of the PPIs found did not have an RNAi phenotype available, causing their scores to be lower. Interactions where only one protein has a severe RNAi phenotype could be promising drug targets, but more RNAi experimentation should be done to determine the RNAi phenotype of the other protein in the interaction pair. For PPI-Indel, nearly all (x of y) the PPIs that ranked highly had a severe RNAi phenotype.

Co-localization of proteins can be considered a prerequisite for direct PPIs. In the absence of specific antibodies to candidate proteins, co-localization of mRNA expression using ISH is an alternative indicator for PPIs. Tissue-specific localization of protein expression in *B. malayi* has been used to confirm gender regulated protein expression and as pointer to protein function[31]. While using the PPI database originating from *C. elegans* to infer PPIs in the parasitic species, we used ISH and synthetic oligonucleotides to study the localization of mRNAs of four pairs of candidate PPIs in two species with very distinct mode of parasitism, the human parasite *B. malayi* and/or the plant-parasite *M. incognita*. This technique allowed tissue-specific localization of the mRNAs of protein pairs in egg cells and developing embryos or in the anterior intestines and pharynx. As part of the development process, targets that yielded promising results via ISH should be tested using alternative techniques such as yeast two-hybrid assay or co-immunoprecipitation to confirm the protein-protein interaction, and biophysical techniques should be used to determine K_D_. Development of a two-hybrid assay for these PPIs also provides a means for screening small molecule drugs that could potentially block the protein-protein interaction.

Certain types of proteins are considered better drug targets than others. Based on Hopkins' work[32], druggable proteins are targets to which drugs that follow Lipinski's rule-of-five bind[33]. Almost half of the targets found by Hopkins et al, fall into six main protein catagories: G-protein coupled receptors (GPCRs), serine/threonine and tyrosine protein kinases, zinc metallopeptidases, serine proteases, nuclear hormone receptors, and phosphodiesterases. For PPI-Nem, the druggability was evaluated, but for the PPI-Indel group, the druggability was added to the scoring function to allow differentiation among a much larger dataset. For PPI-Nem, three of the PPIs had domains that were considered druggable (Table 2 and Table S1 and S4). One would have expected this number to rise for PPI-Indel, considering there were more PPI targets and the scoring function was weighted toward PPIs that were druggable. However, only two PPIs in the top set of PPI-Indel2 had one or more druggable domains (Table 2, Table S1 and S4). The number of druggable domains increased significantly for the PPI-Indel1 group. Interestingly, there are more homooligomeric interactions in PPI-Indel2 versus PPI-Indel1 due to the weighting of the indels in the scoring function. If a protein homooligomerizes and has an indel, it automatically gets a 100 added to its score relative to others, causing the interaction to be ranked higher. Homooligomeric interactions may have evolved to be species specific by changing their interaction surface via indels.

To potentially design drugs for PPIs, either a structure in the PDB or homology to a structure in the PDB is helpful. The database of sequences in the PDB contains information for the entire sequence that was crystallized and tested; the database does not take into account regions that are unresolved in the crystal structure. Therefore, the length of the protein suggested from the primary sequence similarity search may not reflect the model resolved in the X-ray crystal structure. Other studies have used BLASTP to compare the query sequences to the PDB sequence database, not taking the unresolved part of the structure into account when considering homology to the PDB[14], [15]. In the future, running a disorder prediction algorithm, such as DISOPRED[34], on all the sequences would be useful. In general, fewer PPIs for PPI-Nem had homology to the PDB, than those that had RNAi phenotype.

To our knowledge there is only one study on genome-wide drug prioritization in parasitic nematodes[15]. As a test of this methodology, we compared our results to this very different study in which individual proteins were prioritized as drug targets for *B. malayi* [15]. Two of our PPIs from PPI-Nem (O01489/O01489 and Q21234/Q21234) and two proteins from the PPI-Indel group (Q20228/Q9GYK4 and Q20308/Q9U329) were also identified in Kumar et al[15]. The Kumar et al. study did not take into account indels that could be used to uniquely target nematode proteins. Despite the presence of these proteins in the list of the *B. malayi* study, the only way to target these proteins in helminths without also interacting with humans is through differences in sequence homology or indels. In addition to these proteins, there were other proteins in the orthologous groups that overlapped with the set found in the *B. malayi* paper. However, one of the proteins in the PPI lacked homology to the PDB and was not scored. These proteins include: Q93731/Q20775, O76258/O44158, Q20228/Q9GYK4, Q20775/Q93647, P92004/O62215, Q09252/Q965W3, Q20308/Q9U329, O45904/Q9XWD5, O44991/P91318.

Interestingly, the majority of the targets found in our study have expression in the pharynx and intestines. These are excellent sites for drug targeting in helminthes[35], [36]. To determine if this was enrichment for expression in tissue in this study, *C. elegans* tissue expression data was examined. Within the 8474 experiments (see Methods), the following lists the number of experiments where expression in that tissue was seen: 2513 – pharynx, 2808 – muscle, 4218 – neurons, 570 nuclei, and 3157 – intestines. Given that less than 1/3 of the proteins in *C. elegans* have expression in the pharynx, this study significantly enriches for the pharynx tissue with the chi-square goodness of fit test (χ^2^ = 13.41, P < 0.005). The other tissues were not significantly enriched in our study.

Here we provide a unique prospective for prioritizing drug targets for infectious diseases by looking at PPI targets, rather than single proteins. Exploring PPIs as drug targets significantly expands the number of drug targets and provides many new avenues for therapies. Drugs that target PPIs are thought to be the next frontier in therapeutics, and our methodology provides an innovative means of uncovering desperately-needed, novel drug targets for the scourge of parasitic worm infections. As PPI interaction databases and genomic sequences become more available, this approach will provide innovative drug targets for many different parasites and pathogens.

## Methods

### Proteome Databases and Orthologous Groups

The proteomes of various nematodes and hosts were obtained from a number of different sites in 2009 (Table 1). For species where alternative splicing has been found to be present (*B. malayi*, *C. elegans*, *M. incognita*, *H. sapiens*, *A. thaliana)*, only the longest isoform was used in the analysis. Orthologous groups were built using OrthoMCL[37] with default parameters. The orthologous groups were placed in bins, depending on the trophic ecology of the species present in the group (Figure 1 and 2A). For example, there are: bins that include and exclude orthologous groups containing human proteins, bins for the plant parasites and *C. elegans* that include and exclude orthologous groups containing *A. thaliana* proteins, and bins with orthologous groups from the plant parasites and *C. elegans* that exclude orthologous groups containing *A. thaliana* and *H. sapiens* proteins.

### Protein-Protein Interactions

*C. elegans* protein-protein interactions (PPIs) based on experimental interaction evidence from two databases were used: the *C. elegans* Molecular INeraction database (MINT) database[38] (July 2009) with 7,353 PPIs and the IntAct PPI database[39] 10,445 (September 2009). If one of the proteins in the PPI lacked a UniProt ID, the interaction was omitted. A conversion between *C. elegans* gene name and UniProt ID is in Table S12. The *C. elegans* proteins within each bin were compared to the PPI databases, and a PPI was considered a hit if both proteins in the interaction were found within the same bin. When more than one *C. elegans* protein was present in an orthologous group, all *C. elegans* proteins within the orthologous group were mapped to a UniProt ID and then it was determined if a PPI was present. When multiple sequences from other species were in the same orthologous group, they were all mapped to the same UniProtID based on *C. elegans*. Within each taxonomically restricted bin, the IntAct database had more PPIs than MINT.

### Terms in Scoring Function

RNAi phenotypes for *C. elegans* (www.wormbase.org WS195; downloaded on August 2009) were grouped based on Kumar et al[15]. The complete list of RNAi phenotypes sorted by bin is available as Table S13. InterProScan[40] (release 4.5) was run on all the helminth and playhelminth species, and the InterProIDs were compared to the list of InterProIDs considered druggable by Hopkins[32]. The result was incorporated into the scoring function.

To identify similarity to the Protein Data Bank (September 2009), each protein within the different bins was screened using WU-BLASTP (wordmask = seg topcomboN = 1). Only WU-BLASTP hits with percent identity greater than 25, fraction of length greater than 0.5 were considered (Figure 1). All sequence alignments between the orthologous and homologous proteins were done using MUSCLE[41] to further determine the specificity between the sequences found via WU-BLASTP. The PDB homology score was added in only if both proteins had homology to a protein in the PDB. The PDB homology score was based on the best scoring sequence from the orthologous group.

To determine if indels were present, the alignment of nematode sequences and reference sequences was done in a step-wise process for proteins that had orthologs in the host. First, the *C. elegans* sequences from the orthologous groups were extracted, then these sequences were compared against full-length proteins that were publically available in the NCBI database (built 5-26-2009). The WU-BLASTP parameters involved hitdistance = 40 and wordmask = seg, and the results were parsed with a cutoff of 1e^−3^. The sequences were taxonomically restricted to those within Vertebrata and were combined with the *H. sapiens* sequences from the orthologous groups. These sequences will be referred to in this paper as reference sequences for bins where a *H. sapiens* ortholog was considered. For Bin 12, the reference sequences were built by considering sequences taxonomically restricted to Embryophyta. The reference sequences homologous to each *C. elegans* protein from the various orthologous groups were aligned using MUSCLE[41]. The nematode specific sequence profiles from each orthologous group were aligned to the corresponding reference sequence profiles using CLUSTALW[42]. Insertions and deletions specific to nematodes were determined in a method similar to Wang et al [43] and were based on the CLUSTALW profile alignments. Briefly, if the gaps were not present in the reference sequences, the gap was noted as a nematode specific deletion. If there were gaps shared by all reference sequences and no nematode sequences, the sequences were referred to as a nematode-specific insertion. If there were multiple sequences from a single nematode species in an orthologous group, the indel had to be present in at least one sequence within a species. Nematode-specific insertions and deletions were scored within the scoring function.

### Scoring

The following scoring function was used to rank the hits with orthologs in the host:
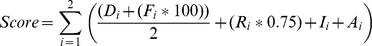
where D = percent sequence identity between query sequence and the sequence of PDB structure; F =  fraction of length between the PDB sequence and query sequence; I  =  50 per protein with an indel; A =  50 per protein considered “druggable” by Hopkins; R = score from *C. elegans* RNAi phenotype bins as listed below: larval/adult lethal/arrest  =  100, embryonic lethal  =  90, sterility  =  80, morphology  =  80, growth  =  70, movement  =  60, vulva  =  50, other  =  10. To balance the PDB homology and RNAi score, the RNAi score was multiplied by 0.75. The scoring broke up the study into three different catagories: PPI-Nem (interactions specific to nematodes), PPI-Indel1 (interactions where one protein had a nematode-specific indel), and PPI-Indel2 (interactions where both proteins had nematode-specific indels). For PPI-Nem, I and A were omitted. If a PPI in PPI-Nem had 100% sequence homology, the entire sequence was present in the PDB, and the RNAi phenotype was larval/adult lethal/arrest, the maximum score that could be achieved is 350. In the case of PPIs in PPI-Indel, a PPI with 100% sequence homolog, the entire sequence present in the PDB, the RNAi phenotype of larval/adult lethal arrest, both proteins considered druggable, and both proteins containing indels would receive a maximum score of 550.

### Expression Profile, Gene Onthology Annotation, and Modelling

Expressed sequence tags (EST) based expression data for *C. elegans*, *T. spiralis*, *B. malayi*, *M. hapla*, *M. incognita* were downloaded from dbEST division of GenBank (September 2009). The ESTs were mapped to the proteins from species they originated from using WU-BLASTX (W = 4, T = 20, B = 1,V = 1,topcomboN = 1) and the expression profile recorded. GO associations of the all helminth and platyhelminth proteins were made by running InterProScan[40] (release 4.5) (Figure 1). Tissue expression for C. elegans was taken from WormMart (www.wormbase.org).

Molecular modeling package (MODELLER 9v7[44]) was used to create homology models of nematode proteins orthologous to Q03601 and Q20329 and their homologs in *H. sapiens* and *A. thaliana* (Figure 4). The PDB template used for homology modeling was chosen using the profile build function in MODELLER. A sequence alignment between the PDB template and individual orthologs of Q03601 and Q20329 in nematodes and homologs in *H. sapiens* and *A. thaliana* was done using the BioInfo metaserver[45]. MODELLER used the sequence alignment from the Meta Server and the template PDB structure (1Q7F for Q03601 and 1D4X for Q20329) to generate five different homology models. The five homology models created by MODELLER were refined using the ClassicRelax protocol in Rosetta3.0[46]. The five models were assessed for quality using their full-atom energy from Rosetta3.0 and two additional programs, Prosa[47] and Molprobity[48], and the best structure was used for subsequent analysis. MODELLER was also used for initial modeling of O01427 using 1FOT and 2JDO. The alignment was done using both MODELLER and the BioInfo metaserver. TASSERLite[49] was ultimately run on O01427 due to incomplete structural resolution for part of the homologous protein in the PDB.

### Fluorescent *In situ* Hybridization (FISH)

When choosing targets for *in situ* testing, the expression in the life stages of *C. elegans* (EST data) and *B. malayi* (microarray expression data (Li, unpublished)) were considered. Ideally, we wanted to test for proteins via FISH that were expressed in the adult worm stages because this is the stage that resides in the host. In addition, the tissue expression in *C. elegans* (www.wormbase.org) and *A. suum* (Mitreva, unpublished) were used to determine if the proteins in the PPI were expressed in the same tissue. Proteins containing indels were checked to ensure alternative splicing did not occur in the indel region of isoforms.

Adult *B. malayi* worms were fixed for 24–72 h in DEPC-treated 4% buffered formaldehyde and embedded in paraffin using standard histological procedures. Sections were deparaffinized and partially digested using pepsin HCl (DakoCytomation, Hamburg, Germany) for approximately 7 minutes and hybridized at 37 °C overnight in a dark humid chamber using 200 ng/ml of custom made biotin (IDT) and digoxygenin (Invitrogen) labeled oligonucleotide probes (*see* Table S14). Probes were checked *in silico* for specificity using BLAST search. A swap of oligonucleotide label was used to ensure that the staining pattern was not affected by the choice of the label. The complementary sense sequence was used as a negative control probe. The hybridization buffer contained 50% formamide, 5XSSC, 0.3 mg/ml yeast tRNA, 100 µg/ml heparin, 1X Denhart's Solution, 0.1% CHAPS and 5mM EDTA. One stringency wash (DakoCytomation) was performed at 42°C for 30 minutes. The slides with hybridized with both antisense (or sense) probes were incubated with 5 µg/ml streptavidin-AlexaFluor 488 conjugate (Invitrogen) and 1 µg/ml anti-digoxigenin-Rhodamin Fab Fragment (Roche) for 30 minutes at room temperature. Both conjugates were diluted in PBS with 0.5 % BSA. Finally sections were rinsed briefly in PBS and covered with a cover slip with ProLong Gold antifade reagent that contains DAPI (Invitrogen). This embedding reagent enables simultaneous fluorescence-based detection of condensed DNA. Sections were examined using a wide field fluorescence microscope (WFFM, Zeiss Axioskop 2 MOT Plus) with a plan-apochromat 100X oil objective or with a Zeiss LSM 510 META (Zeiss, Jena, Germany) confocal laser scanning microcope equipped with a plan-apochromat 63X oil objective and an argon laser for excitation at 488 nm or an HeNe laser for excitation at 543 nm. Confocal Z slices of 0.4 µm were obtained using the Zeiss LSM software.

### In situ hybridization (ISH) in M. incognita

Orthologs of Q03601 (Minc18824) and Q20329 (Minc03587 and Minc058765) were retrieved from the genome of *M. incognita* (http://www.inra.fr/meloidogyne_incognita/). PCR templates for probe synthesis were amplified from L2 first strand cDNAs using gene-specific oligonucleotides (Table S5). DNA sense and anti-sense probes were synthesized by asymmetric PCR using the same oligonucleotides and digoxigenin-labeled dCTP. *In situ* hybridizations were performed as described by Rosso *et al*.[50] Briefly, freshly hatched J2s were fixed in 2% paraformaldehyde for 16 h at 4°C and 4 h at room temperature. Nematodes were cut into sections and permeabilized with proteinase K, acetone and methanol. The sections were hybridized at 37°C with the sense or antisense probe. Nematode sections were incubated in anti-digoxigenin antibody conjugated to alkaline phoshatase. Bound probes were detected by alkaline phosphatase activity staining using NBT (Nitro-Blue Tetrazolium Chloride) /BCIP (5-Bromo-4-Chloro-3'-Indolyphosphate p-Toluidine Salt) substrates.

## Supporting Information

Methods S1 **Additional and more detailed methods.**
(DOC)

Text S1 **Additional discussion on PPI drug targets found in the study.**
(DOC)

Video S1 **Full rotation of a 6 µm section of morula stage embryos labeled for mRNA of Q19126 [XP_00189449.1] (green) and O01427 [XP_001892118.1] (red).**
(MOV)

Figure S1 **Sequence alignment of O01427.** The indels are noted with red boxes.
(DOC)

Figure S2 **A.** **H&E stain of a midbody section of a female *B. malayi* showing the anatomy of the examined parasite sections. **I, intestine; uterus, u; m, morula stage embryos. **B.** Granular staining (arrows) for Q19126 [XP_00189449.1] mRNA in the cytoplasm of morula stage embryos in the midbody region of a female *B. malayi*. Weeker staining was observed in the hypodermis (h) and the uterus epithelium (ue). The biotin labeled probe was detected using AlexaFluor 488-labeled streptavidin (green). **C.** Granular staining (arrows) for O01427 [XP_001892118.1] mRNA in the cytoplasm of egg cells and early morula stage embryos. The biotin labeled probe (label switch) was detected using AlexaFluor 488-labeled streptavidin (green). **D-F.** Confocal laser scanning microscopy (LSM). **D** Granular staining (arrows) for Q19126 [XP_00189449.1] mRNA in the cytoplasm of morulae. The biotin labeled probe was detected using AlexaFluor 488-labeled streptavidin (green). **E.** Identical section as in **D** showing granular staining (arrows) for O01427 [XP_001892118.1] mRNA in the same embyos. The digoxygenin labeled probe was detected using a Rhodamin conjugated anti-digoxygenin antibody (red). **F.** Overlay of **D** and E showing co-localization of expression of both genes. For 3 dimensional rotation of this section see Video S1. **G** Another overlay showing co-localization in morula stage embryos. **H.** Serial section to **E** showing the overlay for both sense probes (no DAPI) indicating the absence of specific labeling. **I.** Co-localization of RNA granules (arrow) positive for P46822 [XP_001895440.1] and Q17581 [XP_001895440.1] in pretzel stage embryos. **J** Pretzel stage embryo at higher magnification showing co-localization (arrows) in a number of granules. **K.** LSM image of co-localization in pretzel stage embryos showing the same pattern (no DAPI). **L.** Serial section to **K**, but hybridized with both sense probes (no DAPI) indicating the absence of specific labeling. Scale bar 10 µm.
(DOC)

Figure S3 **In situ hybridization of Q03601 (Minc18824) and Q20329 (Minc03587 and Minc058765) orthologs on *Meloidogyne incognita* L2.** Transcripts were detected using immunostaining of digoxidenin-labeled antisense probes specific to Minc18824 (A,B) or specific to both Minc03587 and Minc058765 (C,D). For control, in situ hybridizations were performed with the sense Minc18824 (E) and sense Minc03587 -Minc058765 (F) probes. Expression co-localization was evidenced by the presence of the transcripts in the anterior part of the intestine (arrows) and the pharynx (arrow heads). Bar  =  10 mm.
(DOC)

Figure S4 **A. Sequence alignment of Q03601 sequences from orthomcl, as well as homologous proteins from *H. sapiens*.** The secondary structure prediction from the meta server is also shown in the alignment. Blue “E’s” represent beta-sheets, and red “C’s” represent loop regions. Much of the sequence diversity is isolated to the loop regions, thereby creating an accessible method for targeting Q03601 for a nematode specific drug. The loop regions are boxed in red. B. The boxed structures with sequence diversity are mapped to a homology model of Q03601 and highlighted in orange.
(DOC)

Figure S5 ***T. spiralis, C. elegans,* and *H. sapiens* homology models of A. Q03601, B. Q20329, and C. *M. hapla* and *A. thaliana* homology models of Q20329 colored by electrostatic potential in vacuum.** Q03601 did not have any protein sequences in *A. thaliana* with homology to *M. hapla* and *M. incognita*. Although regions of these proteins have homology to *H. sapiens* (A and B) and *A. thaliana* (C), the charges on the surface of *H. sapiens* and *A. thaliana* proteins are different from the charges on the surface of the nematode proteins. Further, orthomcl did not group the *H. sapiens* or *A. thaliana* proteins in the same orthologous groups as the nematode proteins.
(DOC)

Table S1 **Full list of PPI targets in each of the three major groups: specific to nematodes (PPI-Nem), where both proteins contain indels with respect to human host (PPI-Indel2), with one indel with respect to human host (PPI-Indel1).**
(DOC)

Table S2 **PPI-Indel1: Plant parasite PPIs with one indel with respect to Arabidopsis host.** The cutoff score was 399. The following symbols were used to indicate specific features: * indicates druggable, PPIs with + indicate protein with indel, ^a^ RNAi phenotype 1 = Larval/Adult Lethal/Arrest, 2 = Embryonic Lethal, 3 = Sterility, 4 = Morphology, 5 = Growth, 6 = Movement, 7 = Vulva, 8 = Other; ^b^ Indicates analysis group (Nem, Indel2, and Indel1) and also the database where the PPI was found (M = MINT and I = IntAct), ^c^ Stages are listed as L1, L2, L3, L4, egg (Eg), embryo (Em), and Adult (A), ^d^ Localization in *C. elegans* listed as pharynx (P), intestine (I), reproductive (R), muscle (M), hypodermis (H), nervous system (N), somatic (S), embryo (E).
(DOC)

Table S3 **PPI-Indel2: Plant parasite PPIs where both proteins contain indels with respect to Arabidopsis host.** The cutoff score was 393. The following symbols were used to indicate specific features: * indicates druggable, PPIs with + indicate protein with indel, ^a^ RNAi phenotype 1 = Larval/Adult Lethal/Arrest, 2 = Embryonic Lethal, 3 = Sterility, 4 = Morphology, 5 = Growth, 6 = Movement, 7 = Vulva, 8 = Other; ^b^ Indicates analysis group (Nem, Indel2, and Indel1) and also the database where the PPI was found (M = MINT and I = IntAct), ^c^ Stages are listed as L1, L2, L3, L4, egg (Eg), embryo (Em), and Adult (A), ^d^ Localization in *C. elegans* listed as pharynx (P), intestine (I), reproductive (R), muscle (M), hypodermis (H), nervous system (N), somatic (S), embryo (E).
(DOC)

Table S4 **The total score broken down into terms for the full list of PPI targets** in each of the three major groups: specific to nematodes (PPI-Nem), where both proteins contain indels with respect to human host (PPI-Indel2), with one indel with respect to human host (PPI-Indel1). The score is broken down into the terms in the scoring function, so that the PPI can be evaluated for further investigation.
(DOC)

Table S5 **Oligonucleotide probes used for ISH.** Sense probes with the dogoxigenin label were used as controls for each probe.
(DOC)

Table S6 **Taxonomically restricted orthologous groups.**
(DOC)

Table S7 **PPIs from MINT and IntAct (PPI-Nem).**
(DOC)

Table S8 **PPIs from MINT and IntAct (PPI-Indel).**
(DOC)

Table S9 **PPI-Nem: Unique protein-protein interactions in each bin found from the MINT and IntAct Databases.** Interactions in bold were found in both the MINT and IntAct Databases.
(DOC)

Table S10 **PPI-Nem: Unique protein-protein interactions in each bin found from the MINT and IntAct Databases where both proteins involved in the protein-protein interaction had an RNAi phenotype.** Interactions in bold were found in both the MINT and IntAct Databases.
(DOC)

Table S11 **PPI-Nem: PPIs in which one of the proteins has an RNAi phenotype.** These proteins might be good targets for subsequent RNAi experiments. Interactions in bold were found in both the MINT and IntAct Databases.
(DOC)

Table S12 **Conversion of UniprotIDs to C. elegans gene IDs.**
(DOC)

Table S13 **Complete list of RNAi phenotypes sorted by bin.**
(DOC)

Table S14 **Oligonucleotide probes used for FISH.** Sense probes with the respective label were used as controls for each probe.
(DOC)
