# Supplementary material for: Targeting Protein-Protein Interactions for Parasite Control
Source: PLoS One. 2011 Apr 27;6(4):e18381. doi: 10.1371/journal.pone.0018381 (PMC3083401; doi:10.1371/journal.pone.0018381)
Supplement: Methods S1 — Additional and more detailed methods. (DOC) [file pone.0018381.s001.doc]

**Proteome Databases**

The proteomes of various nematodes and hosts were obtained from a number of different sites in 2009 (Table 1). For species where alternative splicing has been found to be present (*B. malayi*, *C. elegans*, *M. incognita*, *H. sapiens*, *A. thaliana)*, only the longest isoform was used in the analysis (Table 1).

**Assigning Orthologous Groups**

Orthologous groups were built using OrthoMCL[1] with default parameters. The orthologous groups were placed in bins, depending on the trophic ecology of the species present in the group (Figure 1 and 2A). These bins were generated considering major differences among studied parasites. For example, there are: bins that include and exclude orthologous groups containing human proteins, bins for the plant parasites and *C. elegans* that include and exclude orthologous groups containing *A. thaliana* proteins, and bins with orthologous groups from the plant parasites and *C. elegans* that exclude orthologous groups containing *A. thaliana* and *H. sapiens* proteins.

**Protein-Protein Interactions**

*C. elegans* protein-protein interactions (PPIs) based on experimental interaction evidence from two databases were used: the *C. elegans* Molecular INeraction database (MINT) database (July 2009) with 7,353 PPIs[2] and the IntAct PPI database 10,455 PPIs (September 2009[3]). If one of the proteins in the PPI lacked a UniProt ID, the interaction was omitted. A conversion between *C. elegans* gene name and UniProt ID is in Table S12. The *C. elegans* proteins within each bin were compared to the PPI databases, and a PPI was considered a hit if both proteins in the interaction were found within the same bin. When more than one *C. elegans* protein was present in an orthologous group, all *C. elegans* proteins within the orthologous group were mapped to a UniProt ID and then it was determined if a PPI was present. When multiple sequences from other species were in the same orthologous group, they were all mapped to the same UniProtID based on *C. elegans*. Within each taxonomically restricted bin, the IntAct database had more PPIs than MINT.

**RNAi Phenotype and Druggability**

RNAi phenotypes for *C. elegans* ([www.wormbase.org](https://gscmail.wustl.edu/squirrelmail/src/www.wormbase.org) WS195; downloaded on August 2009) were grouped based on Kumar et al[4]. The complete list of RNAi phenotypes sorted by bin are available as Table S13. Hopkins et al developed a list of proteins with certain InterPro IDs that are considered druggable[5]. InterProScan[6] (release 4.5) was run on all the helminth and playhelminth species, and the InterProIDs were compared to the list of InterProIDs considered druggable by Hopkins; the result was incorporated into the scoring function.

**Homology to the Protein Data Bank**

To identify similarity to the Protein Data Bank (September, 2009) each protein within the different bins was screened using WU-BLASTP (wordmask=seg topcomboN=1). Only WU-BLASTP hits with percent identity greater than 25, fraction of length greater than 0.5 were considered, and an e-value less than 1e-4 (Figure 1). All sequence alignments between the orthologous and homologous proteins were done using MUSCLE[7] to further determine the specificity between the sequences found via WU-BLASTP. The PDB homology score was added in only if both proteins had homology to a protein in the PDB. The PDB homology score was based on the best scoring sequence from the orthologous group.

**Indel Detection**

The alignment of nematode sequences and reference sequences was done in a step-wise process for proteins that had orthologs in the host. First, the *C. elegans* sequences from the orthologous groups were extracted, then these sequences were compared against full-length proteins that were publically available in the NCBI database (built 5-26-2009). The WU-BLASTP parameters involved hitdistance=40 and wordmask=seg, and the results were parsed with a cutoff of 1.0e-3. The sequences were taxonomically restricted to those within Vertebrata and were combined with the *H. sapiens* sequences from the orthologous groups. These sequences will be referred to in this paper as reference sequences for bins where a *H. sapiens* ortholog was considered. For Bin 12, the reference sequences were built by considering sequences taxonomically restricted to Embryophyta. The reference sequences homologous to each *C. elegans* protein from the various orthologous groups were aligned using MUSCLE[7]. The nematode specific sequence profiles from each orthologous group were aligned to the corresponding reference sequence profiles using CLUSTALW[8]. Insertions and deletions specific to nematodes were determined in a method similar to Wang et al[9] and were based on the CLUSTALW profile alignments. Briefly, if the gaps were not present in the reference sequences, the gap was noted as a nematode-specific deletion. If there were gaps shared by all reference sequences and no nematode sequences, the sequences were referred to as a nematode-specific insertion. If there were multiple sequences from a single nematode species in an orthologous group, the indel had to be present in at least one sequence within a species. Nematode-specific insertions and deletions were scored within the scoring function.

**Scoring**

$Score=\sum_{i=1}^{2} \left(\frac{(D_{i}+(F_{i}*100)}{2}+(R_{i}*0.75)+I_{i}+A_{i}\right)$The following scoring function was used to rank the hits with orthologs in the host:

where D=percent sequence identity between query sequence and the sequence of PDB structure; F= fraction of length between the PDB sequence and query sequence; I = 50 per protein with an indel; A= 50 per protein considered “druggable” by Hopkins; R=score from *C. elegans* RNAi phenotype bins as listed below: larval/adult lethal/arrest = 100, embryonic lethal = 90, sterility = 80, morphology = 80, growth = 70, movement = 60, vulva = 50, other = 10. To balance the PDB homology and RNAi score, the RNAi score was multiplied by 0.75. The scoring broke up the study into three different catagories: PPI-Nem (interactions specific to nematodes), PPI-Indel1 (interactions where one protein had a nematode-specific indel), and PPI-Indel2 (interactions where both proteins had nematode-specific indels). For PPI-Nem, I and A were omitted. If a PPI in PPI-Nem had 100% sequence homology, the entire sequence was present in the PDB, and the RNAi phenotype was larval/adult lethal/arrest, the maximum score that could be achieved is 350. In the case of PPIs in PPI-Indel, a PPI with 100% sequence homolog, the entire sequence present in the PDB, the RNAi phenotype of larval/adult lethal arrest, both proteins considered druggable, and both proteins containing indels would receive a maximum score of 550.

**Expression Profile and Gene Ontology Annotation**

Expressed sequence tags (EST) based expression data for *C. elegans*, *T. spiralis*, *B. malayi*, *M. hapla*, *M. incognita* were downloaded from dbEST division of GenBank (Sept., 2009). The ESTs were mapped to the proteins from species they originated from using WU-BLASTX (W=4, T=20, B=1,V=1,topcomboN=1) and expression profile recorded. GO associations of the all helminth and platyhelminth proteins were made by running InterProScan[6] (release 4.5) (Figure 1).

**Homology Modeling and de novo Structure Prediction**

Molecular modeling package (MODELLER 9v7[10]) was used to create homology models of nematode proteins orthologous to Q03601 and Q20329 and their homologs in *H. sapiens* and *A. thaliana* (Figure 4 and Text S1). The PDB template used for homology modeling was chosen using the profile build function in MODELLER. A sequence alignment between the PDB template and individual orthologs of Q03601 and Q20329 in nematodes and homologs in *H. sapiens* and *A. thaliana* was done using the BioInfo metaserver[11]. MODELLER used the sequence alignment from the Meta Server and the template PDB structure (1Q7F for Q03601 and 1D4X for Q20329) to generate five different homology models. The five homology models were refined using the ClassicRelax protocol in Rosetta3.0[12-14]. The five models were assessed for quality using their full-atom energy from Rosetta3.0 and two additional programs, Prosa[15] and Molprobity[16], and the best structure was used for subsequent analysis. MODELLER was also used for initial modeling of O01427 using 1FOT and 2JDO. The alignment was done using both MODELLER and the BioInfo metaserver. TASSERLite[17] was ultimately run on O01427 due to incomplete structural resolution for part of the protein in the PDB.

**Fluorescent *In situ* Hybridization (FISH)**

When choosing targets for *in situ* testing, the expression in the life stages of *C. elegans* and *B. malayi* were considered. The microarray expression information was used to determine if the proteins in the PPI were expressed at the same life stage (Li, unpublished). Ideally, we wanted to test for proteins via *FISH* that were expressed in the adult worm stages because this is the stage that resides in the host. In addition, the tissue expression in *C. elegans* (www.wormbase.org) and *A. suum* (Mitreva, unpublished) were used to determine if the proteins in the PPI were expressed in the same tissue.

Adult *B. malayi* worms were fixed for 24-72 h in DEPC-treated 4% buffered formaldehyde and embedded in paraffin using standard histological procedures. Sections were deparaffinized and partially digested using pepsin HCl (DakoCytomation, Hamburg, Germany) for approximately 7 minutes and hybridized at 37 °C overnight in a dark humid chamber using 200 ng/ml of custom made biotin (IDT) and digoxygenin (Invitrogen) labeled oligonucleotide probes (*see* Table S14). Probes were checked *in silico* for specificity using BLAST search. A swap of oligonucleotide label was used to ensure that the staining pattern was not affected by the choice of the label. The complementary sense sequence was used as a negative control probe. The hybridization buffer contained 50% formamide, 5XSSC, 0.3 mg/ml yeast tRNA, 100 µg/ml heparin, 1X Denhart’s Solution, 0.1% CHAPS and 5mM EDTA. One stringency wash (DakoCytomation) was performed at 42 °C for 30 minutes. The slides with hybridized with both antisense (or sense) probes were incubated with 5 µg/ml streptavidin-AlexaFluor 488 conjugate (Invitrogen ) and 1 µg/ml anti-digoxigenin-Rhodamin Fab Fragment (Roche) for 30 minutes at room temperature. Both conjugates were diluted in PBS with 0.5 % BSA. Finally sections were rinsed briefly in PBS and covered with a cover slip with ProLong Gold antifade reagent that contains DAPI (Invitrogen). This embedding reagent enables simultaneous fluorescence-based detection of condensed DNA. Sections were examined **using a wide field fluorescence microscope (WFFM,** Zeiss Axioskop 2 MOT Plus) with a plan-apochromat 100X oil objective or with a Zeiss LSM 510 META (Zeiss, Jena, Germany) confocal laser scanning microcope equipped with a plan-apochromat **63X oil objective and an argon laser for excitation at 488 nm or an HeNe laser for excitation at 543 nm. Confocal Z slices of 0.4 µm were obtained using the Zeiss LSM software.**

***In situ* hybridization (ISH) in *M. incognita***

Orthologs of Q03601 (Minc18824) and Q20329 (Minc03587 and Minc058765) were retrieved from the genome of *M. incognita* (http://www.inra.fr/meloidogyne_incognita/). PCR templates for probe synthesis were amplified from L2 first strand cDNAs using gene-specific oligonucleotides (Table S5). DNA sense and anti-sense probes were synthesized by asymmetric PCR using the same oligonucleotides and digoxigenin-labeled dCTP. *In situ* hybridizations were performed as described by Rosso *et* *al*.[18]. Briefly, freshly hatched J2s were fixed in 2% paraformaldehyde for 16 h at 4°C and 4 h at room temperature. Nematodes were cut into sections and permeabilized with proteinase K, acetone and methanol. The sections were hybridized at 37 °C with the sense or antisense probe. Nematode sections were incubated in anti-digoxigenin antibody conjugated to alkaline phoshatase. Bound probes were detected by alkaline phosphatase activity staining using NBT (Nitro-Blue Tetrazolium Chloride) /BCIP (5-Bromo-4-Chloro-3'-Indolyphosphate p-Toluidine Salt) substrates.

**References**

1. Li, L., C.J. Stoeckert, Jr., and D.S. Roos (2003) *OrthoMCL: identification of ortholog groups for eukaryotic genomes.* Genome Res. **13**(9): 2178-89.

2. Ceol, A., A. Chatr Aryamontri, L. Licata, D. Peluso, L. Briganti, et al. (2009) *MINT, the molecular interaction database: 2009 update.* Nucleic Acids Res.

3. Aranda, B., P. Achuthan, Y. Alam-Faruque, I. Armean, A. Bridge, et al. (2009) *The IntAct molecular interaction database in 2010.* Nucleic Acids Res.

4. Kumar, S., K. Chaudhary, J.M. Foster, J.F. Novelli, Y. Zhang, et al. (2007) *Mining predicted essential genes of Brugia malayi for nematode drug targets.* PLoS One. **2**(11): e1189.

5. Hopkins, A.L. and C.R. Groom (2002) *The druggable genome.* Nat Rev Drug Discov. **1**(9): 727-30.

6. Hunter, S., R. Apweiler, T.K. Attwood, A. Bairoch, A. Bateman, et al. (2009) *InterPro: the integrative protein signature database.* Nucleic Acids Res. **37**(Database issue): D211-5.

7. Edgar, R.C. (2004) *MUSCLE: multiple sequence alignment with high accuracy and high throughput.* Nucleic Acids Res. **32**(5): 1792-7.

8. Thompson, J.D., D.G. Higgins, and T.J. Gibson (1994) *CLUSTAL W: improving the sensitivity of progressive multiple sequence alignment through sequence weighting, position-specific gap penalties and weight matrix choice.* Nucleic Acids Res. **22**(22): 4673-80.

9. Wang, Z., J. Martin, S. Abubucker, Y. Yin, R.B. Gasser, et al. (2009) *Systematic analysis of insertions and deletions specific to nematode proteins and their proposed functional and evolutionary relevance.* BMC Evol Biol. **9**: 23.

10. Sali, A. and T.L. Blundell (1993) *Comparative protein modelling by satisfaction of spatial restraints.* J Mol Biol. **234**(3): 779-815.

11. Ginalski, K., A. Elofsson, D. Fischer, and L. Rychlewski (2003) *3D-Jury: a simple approach to improve protein structure predictions.* Bioinformatics. **19**(8): 1015-8.

12. Bradley, P., K.M. Misura, and D. Baker (2005) *Toward high-resolution de novo structure prediction for small proteins.* Science. **309**(5742): 1868-71.

13. Rohl, C.A., C.E. Strauss, K.M. Misura, and D. Baker (2004) *Protein structure prediction using Rosetta.* Methods Enzymol. **383**: 66-93.

14. Schueler-Furman, O., C. Wang, P. Bradley, K. Misura, and D. Baker (2005) *Progress in modeling of protein structures and interactions.* Science. **310**(5748): 638-42.

15. Wiederstein, M. and M.J. Sippl (2007) *ProSA-web: interactive web service for the recognition of errors in three-dimensional structures of proteins.* Nucleic Acids Res. **35**(Web Server issue): W407-10.

16. Davis, I.W., A. Leaver-Fay, V.B. Chen, J.N. Block, G.J. Kapral, et al. (2007) *MolProbity: all-atom contacts and structure validation for proteins and nucleic acids.* Nucleic Acids Res. **35**(Web Server issue): W375-83.

17. Zhang, Y. and J. Skolnick (2004) *Automated structure prediction of weakly homologous proteins on a genomic scale.* Proc Natl Acad Sci U S A. **101**(20): 7594-9.

18. Rosso, M.N., B. Favery, C. Piotte, L. Arthaud, J.M. De Boer, et al. (1999) *Isolation of a cDNA encoding a beta-1,4-endoglucanase in the root-knot nematode Meloidogyne incognita and expression analysis during plant parasitism.* Mol Plant Microbe Interact. **12**(7): 585-91.
