# Supplementary material for: Targeting Protein-Protein Interactions for Parasite Control
Source: PLoS One. 2011 Apr 27;6(4):e18381. doi: 10.1371/journal.pone.0018381 (PMC3083401; doi:10.1371/journal.pone.0018381)
Supplement: Text S1 — Additional discussion on PPI drug targets found in the study. (DOC) [file pone.0018381.s002.doc]

***Potential Drug Targets – Unique to Nematodes (PPI-Nem)***

The majority of the protein-protein interaction drug targets that emerged from this study had a UniProt classification of zinc finger, integrase, or serine-kinase (Table 2, Main Text). Nuclear hormone receptors have been found to make good drug targets in general[1] and for parasitic nematodes[2,3]. The interaction between the *C. elegans* proteins, Q03601/Q20329, is the highest ranked PPI target and was found in the IntAct database. Typically, proteins in the NHL-1 family are involved in protein ubiquitination[4]. The PPI between Q03601 and Q20329 was present in bins 22, 18, and 14. To test if these proteins were nematode specific proteins, BLASTP was run on all the sequences and compared to the *H. sapiens* and *A. thaliana* genomes. Although there were no *H. sapiens* or *A. thaliana* proteins present in the orthologous groups assigned by OrthoMCL, some of the proteins still had homologs in their respective hosts. Obviously there must be substantial differences between the worm and host proteins for them not to be placed in the same orthologous group. Q03601 is a very large protein in *B. malayi*, *C. elegans*, and *T. spiralis*. The N-terminal region is dramatically reduced in *H. sapiens* and is simply not present in *M. hapla* and *M. incognita*. In all the proteins, the C-terminal end has homology to a beta-propeller in the PDB. To determine if the beta-propeller region of the worm and host proteins were different enough to design a specific drug, homology models of the C-terminal region were modeled and the electrostatics in vacuum were calculated using PyMOL[5]. The results of the electrostatic calculations are shown in Figure 4 and S5. There are charged patches on the nematodes that are not present on the hosts. Further, there is some difference between the nematodes, which also provides an opportunity for specific drug targeting between nematodes. The N-terminal region has no homology to any protein in the PDB, but offers an interesting method to target the nematode specific proteins. There is no homologous protein in *A. thaliana*, making this a particularly interesting target for plant nematodes. Further a sequence alignment and secondary structure alignment between the hosts and nematodes shows the beta sheet regions have much more sequence homology than the loop regions (Figure S4). Q20329 was modeled in the same way for the proteins in all the species, and the electrostatic charge was calculated using PyMOL. Again, the surface presents very different charges between the nematodes and hosts (Figure S5).

The tissue expression data available for the *C. elegans* ([http://www.wormbase.org](http://www.wormbase.org/)) ortholog of Q03601 is localized in the pharynx and intestine. A recent study (Mitreva, unpublished) which used microarray technology to look at the tissue expression in *A. suum*, confirmed that the Q03601 ortholog was differentially expressed in the intestines of males and females and the pharynx of males, whereas the Q20329 ortholog was differentially expressed in the pharynx and intestines of males and females. In *B. malayi*, both proteins are differentially expressed in the microfilarial and 12 week female stages. Q20329 is also differentially expressed in 6 week males and females, L3, and L4 life stages. Given these proteins are expressed in similar life stages and in the same tissue types, it is likely that these proteins do interact as suggested by the initial paper that reported the PPI[6]. Expression of Q03601 and Q20329 orthologs in the plant parasite *M. incognita* was analyzed via *in situ* hybridization. Expression of the genes clearly co-localized in the anterior part of the intestine in L2 stage. In addition, symmetrical signals aligned in two rows indicated possible co-localization in muscle cells of the pharynx (Figure S3).

***Zinc Finger Targets***

A hit in Bin 14, the homodimeric interaction of O01489[7] is also a zinc finger target and was found in both the MINT and IntAct databases. According to its InterPro classification (IPR001878), it falls into the zinc knuckle classification. This protein is a hit from the *B. malayi* paper, but does not have any homology to a protein in the PDB. The RNAi phenotype of O01489 is classified as embryonic lethal arrest. The protein is expressed in nearly all *C. elegans*  lifestages: egg, embryo, L1, L2, L4, and it is expressed in all *B. malayi* lifestages tested: microfiliarial, 6 week male and female, 12 week male and female, L3, and L4. The protein is also expressed in the following tissues, some of which are good tissues for drug targeting[8,9], including pharynx, intestine, reproductive tissue, and the nervous system in *C. elegans*.

Found in Bin 14, protein O45666 (NHR-49) is classified as a nuclear hormone receptor (IPR001628, IPR008946,IPR000536) and interacts with itself (MINT database[10]) and with Q09528 (NHR-19 IPR013088). The two PPIs, O45666/O45666 and O45666/Q09528, were present in both the MINT and IntAct databases. Both proteins are considered druggable domains by Hopkins et al[1]. Nuclear hormone receptors have been identified as fruitful targets for *B. malayi* drugs because they have been found to be involved in essential processes such as molting[2,3]. The RNAi phenotypes for O45666 are larval/adult lethal/arrest, embryonic lethal, growth, and movement, and Q09528 does not have an RNAi phenotype. The two proteins are expressed in the same lifestages: L1, L4, and adult, and O45666 is also expressed in L2 and the embryo. The proteins in both interactions could be modeled and a drug could potentially be designed based on the structure, as they have homology to proteins in the PDB. Based on the *A. suum* microarray data, O45666 is differentially expressed in the ovaries, male and female intestines, seminal vesicles, and testes (Mitreva, unpublished).

***Integrase Target***

The protein-protein interaction between Q21234/Q21234 was found using both the IntAct and MINT databases in Bin 22. The InterPro ID, IPR001584, indicated that this protein is an integrase, but also may contain a zinc finger motif (IPR001878). The protein was also designated as a hit in the drug prioritization study for *B. malayi*[11] and is considered a druggable domain by Hopkins et al[1]. Although Q21234 does not have homology to any proteins in the PDB, it has a strong RNAi phenotype, consisting of larval/adult lethal/arrest, embryonic lethal/arrest, sterility, and growth defect in *C. elegans*. In *C. elegans*, the protein is expressed in the embryo, L1, and L4 stages, and in *B. malayi*, the protein is expressed in the microfilarial, 6 week female, 12 week male and female, L3, and L4 stages (Li, unpublished). Based on *A. suum* expression data, Q21234 is differentially expressed in the female and male pharynx and intestine, seminal vesicle, testis, uterus, ovaries, and head (Mitreva, unpublished), which are good locations for drug targeting[8,9].

***Serine-kinase Target***

Found via the IntAct database in Bin 22, the proteins in the PPI between Q8MYQ1/Q22631 are thought to be a serine/thr-kinase and thrombospondin, respectively. Although the InterProID is not listed by Hopkins et al[1], serine/thr kinases are considered good drug targets[1]. In *C. elegans*, both proteins are expressed in the L1 lifestage, but Q22631 is also expressed in the embryo, L2, and L4 stages. Both proteins have homology to proteins in the PDB, but no RNAi phenotype has been observed for Q22631. Q8MYQ1 has a vulva/egg laying phenotype, based on RNAi experiments.

**Potential Drug Targets – Indel Targeting (PPI-Indel)**

***PPI-Indel1***

Chosen because of its expression in several life stages in *B. malayi*, the interaction between Q95005 and Q19207 was ranked 4^th^ among PPIs where one protein had an indel and was also found in Bins 3, 7, 17, and 21. Both proteins make a large number of PPIs within both the MINT and IntAct Databases. Q95005 has 26 interactions in both MINT and IntAct, and Q19207 has 10 interactions in MINT and 5 in IntAct. Both genes have severe RNAi phenotypes in *C. elegans*, including embryonic lethal, larval/adult lethal arrest, sterility, growth and movement. In addition, Q19207 also has a morphology defect in some RNAi experiments and also is a considered a druggable domain by Hopkins. Q95005 has very good homology to proteins in the PDB (100% over 99% of the length), whereas Q19207 has very good homology but over only 50% of its length. Q19207 has two deletions relative to vertebrate genomes. In the *A. suum* microarray, both proteins were differentially expressed in the male and female head, and Q95005 was found to be expressed in body wall muscle in larvae, as well as pharynx, anal depressor muscle, and body wall muscle. Both proteins were expressed in the micofiliarial stage, L3, L4 (2wk), and L5 (12 wk) stages, and Q95005 was also expressed in the L4 (6wk) stage. In *C. elegans*, both proteins were expressed in embryo, L1, and adult. Q95005 was also expressed in L4 and Q19207 in egg and L2. Q95005 encodes a subunit of the core 20S proteasome complex (IPR001353, IPR000426), which is involved in protein degradation pathways. Q19207 encodes 3-hydroxy-3-methyl-glutaryl-CoA reductase (HMG-CoA reductase) (IPR009029, IPR002202), which is involved in the mevalonate pathway that eventually produces cholesterol. This PPI was found via yeast two hybrid[12], and co-localization of expression in eggs and developing embryos was shown via *in situ* hybridization in *B. malayi* (data not shown).

Ranked 12^th^ out of all PPIs with one indel and also promising due to both proteins being expressed in several life stages in *B. malayi*, Q22799 and Q93572, both proteins have severe RNAi phenotypes in *C. elegans*, including embryonic lethal arrest, larval/adult lethal arrest, and sterility. Additional RNAi phenotypes for Q22799 include growth, movement, morphology, and vulva defect. The extremely severe RNAi phenotypes for Q22799 are not surprising given the large number of PPIs in the databases for this protein (74 MINT and 51 IntAct), indicating protein is a critical hub. Q93572 also makes many interactions (14 MINT). Both proteins have good homology to the PDB, with Q22799 and Q93572 having 100% homology over 98% and 99% of length. Q93572 has one deletion relative to vertebrate genomes. Although these proteins were not on the *B. malayi* microarray experiment, both proteins are expressed in the adult stage in *C. elegans*, and Q93572 is also expressed in L1, L2, L3, L4, and embryo stages. Both proteins are expressed in similar tissue in *C. elegans*, including pharynx, muscle wall, and intestine. These sites are good for drug targeting. In *A. suum*, the proteins were differentially expressed in the testis and male intestines. This PPI was found in Bins 7 and 21, but the indel was only in Bin 7. Q22799 encodes a dynein light chain (IPR001372), which is involved in transport from the microtubule plus to minus end. Q93572 encodes part of the 60S portion of the ribosome (IPR001813). This PPI was also found via yeast two hybrid[13].

***PPI-Indel2***

Having the most promising expression in many life stages in *B. malayi*, the interaction between P46822 and Q17581 was ranked 7th highest in the PPI-Indel2 group, with P46822 having an insertion and Q17581 having a deletion. The interaction was found in Bin 21 in both MINT and IntAct. Neither domain had an InterProID that correlated with InterProIDs found to be druggable by Hopkins, but both proteins have a severe embryonic lethal RNAi phenotype. In addition, RNAi has also yielded additional phenotypes for Q17581 in *C. elegans*, including sterility, larval adult lethal arrest, growth and morphology defects. Over 80% of the proteins in this group have homology to a protein in the PDB with a sequence identity of 78.6% and 50.8% for P46822 and Q17581, respectively. The proteins are expressed in 3 of the same life stages in *C. elegans*, including embryo, L1, and adult. P46822 is also expressed in the egg and L4 stages in *C. elegans*. In *B. malayi*, the proteins were expressed in all the life stages that were present on the microarray (Li, unpublished). In protein interaction networks, proteins that make many PPIs are considered more essential than proteins that make a small number of interactions. P46822 is a very well connected hub in the PPI interaction network, with 20 and 21 PPIs in MINT and IntAct, respectively. As a kinesin light chain, P46822 makes many PPIs in the cell to carry out its function and this is reflected in the number of PPIs emanating from this hub with 20 and 21 PPIs in MINT and IntAct, respectively. Each kinesin protein has two heavy chains and two light chains. Typically moving from the minus end to the plus end of a microtubule, the kinesin light chain binds to the cargo being transported. Q17581 makes 2 PPIs in MINT and IntAct. The NCBI KOG (clusters of euKaryotic Orthologous Groups) classifies this protein as a protein CELTIX-1 protein containing a bromodomain that binds to IRF-2, a transcription factor. The bromodomain specifically recognizes acetylated lysines. This interaction was found via yeast two hybrid [13] and co-localization of expression in the pretzel stage of the developing embryo was also found using FISH (Figure S2) and a complementary *in situ* technique.

***Co-localization of protein expression as prerequisite and indicator of PPI***

In contrast to the model nematode *C. elegans* genetic manipulations such as RNAi are not widely established for animal parasitic nematodes[14,15]. Although the filarial nematode *B. malayi* was the first parasitic nematode for which a draft genome was available[2] the lack of tools for functional genomics impedes experimental confirmation of PPIs in this species. FISH and synthetic oligonucleotides were used to study the localization of mRNAs of two pairs of candidate PPIs in *B. malayi*. This technique not only allows the tissue specific, but also the subcellular, localization of mRNA and the hybridization of two differently labeled anti-sense probes to the same worm section, enabling simultaneous detection of both messages. We observed a highly tissue specific expression of both protein pairs especially in egg cells and developing embryos. In positive cells, mRNA was not evenly distributed within the cytoplasm, but confined to distinct granules, which have not been described in filarial nematodes before. In *C. elegans* different types of RNA granules have been described such as processing bodies, germ granules and stress granules. Although these RNA/protein complexes have different functions such as storage for RNA splicing or for RNA degradation they may also interact[16]. Due to the absence of specific markers for RNA granules in *B. malayi* we were not able to determine the type of RNA granules to which our probes hybridize. Although probes for both pairs of candidate PPIs hybridized to targets in the same cell, localization to the same granule was observed only occasionally (Fig 3F, Figure S2 F, G, I-K). However, localization of interacting proteins and their messages in the same cell may be more a important prerequisite for PPIs rather than the localization of messages in the same RNA granule type, because dynamics of RNA splicing or RNA degradation may vary for interacting proteins.

In the absence of specific antibodies, ISH allows tissular localization of gene expression. We used gene-specific antisense probes to localize transcripts from Q20329 and Q03601 orthologs in the plant parasite M*. incognita*. One ortholog for Q03601 (Minc18824) and two orthologs for Q20329 (Minc03587 and Minc058765) were analyzed. Sequence conservation between Minc03587 and Minc058765allowed the design of an anti-sense probe specific to both Q20329 orthologs. Although Minc03587 and Minc058765 were highly similar, a 174 bp insertion in Minc03587 allowed the synthesis of a specific probe for this gene. ISH indicated active transcription of the genes in the anterior part of the intestine, providing evidence for expression co-localization. In addition, labeling in the pharynx was observed for the three genes analyzed that could possibly be localized in pharyngeal muscle cells (Figure S3).

**References**

1. Hopkins AL, Groom CR (2002) The druggable genome. Nat Rev Drug Discov 1: 727-730.

2. Ghedin E, Wang S, Spiro D, Caler E, Zhao Q, et al. (2007) Draft genome of the filarial nematode parasite Brugia malayi. Science 317: 1756-1760.

3. Scott AL, Ghedin E (2009) The genome of Brugia malayi - all worms are not created equal. Parasitol Int 58: 6-11.

4. Matthews JM, Sunde M (2002) Zinc fingers--folds for many occasions. IUBMB Life 54: 351-355.

5. DeLano W (2002) The PyMOL Molecular Graphics System. DeLano Scientific, Palo Alto, CA, USA.

6. Simonis N, Rual JF, Carvunis AR, Tasan M, Lemmens I, et al. (2009) Empirically controlled mapping of the Caenorhabditis elegans protein-protein interactome network. Nat Methods 6: 47-54.

7. Walhout AJ, Reboul J, Shtanko O, Bertin N, Vaglio P, et al. (2002) Integrating interactome, phenome, and transcriptome mapping data for the C. elegans germline. Curr Biol 12: 1952-1958.

8. Mitreva M, Zarlenga DS, McCarter JP, Jasmer DP (2007) Parasitic nematodes - from genomes to control. Vet Parasitol 148: 31-42.

9. van den Enden E (2009) Pharmacotherapy of helminth infection. Expert Opin Pharmacother 10: 435-451.

10. Ceol A, Chatr Aryamontri A, Licata L, Peluso D, Briganti L, et al. (2009) MINT, the molecular interaction database: 2009 update. Nucleic Acids Res.

11. Kumar S, Chaudhary K, Foster JM, Novelli JF, Zhang Y, et al. (2007) Mining predicted essential genes of Brugia malayi for nematode drug targets. PLoS One 2: e1189.

12. Boxem M, Maliga Z, Klitgord N, Li N, Lemmens I, et al. (2008) A protein domain-based interactome network for C. elegans early embryogenesis. Cell 134: 534-545.

13. Li S, Armstrong CM, Bertin N, Ge H, Milstein S, et al. (2004) A map of the interactome network of the metazoan C. elegans. Science 303: 540-543.

14. Viney ME, Thompson FJ (2008) Two hypotheses to explain why RNA interference does not work in animal parasitic nematodes. Int J Parasitol 38: 43-47.

15. Knox DP, Geldhof P, Visser A, Britton C (2007) RNA interference in parasitic nematodes of animals: a reality check? Trends Parasitol 23: 105-107.

16. Gallo CM, Munro E, Rasoloson D, Merritt C, Seydoux G (2008) Processing bodies and germ granules are distinct RNA granules that interact in C. elegans embryos. Dev Biol 323: 76-87.
