# Supplementary figures and images for: Targeting Protein-Protein Interactions for Parasite Control
Source: PLoS One. 2011 Apr 27;6(4):e18381. doi: 10.1371/journal.pone.0018381 (PMC3083401; doi:10.1371/journal.pone.0018381)

**Figure S1.**


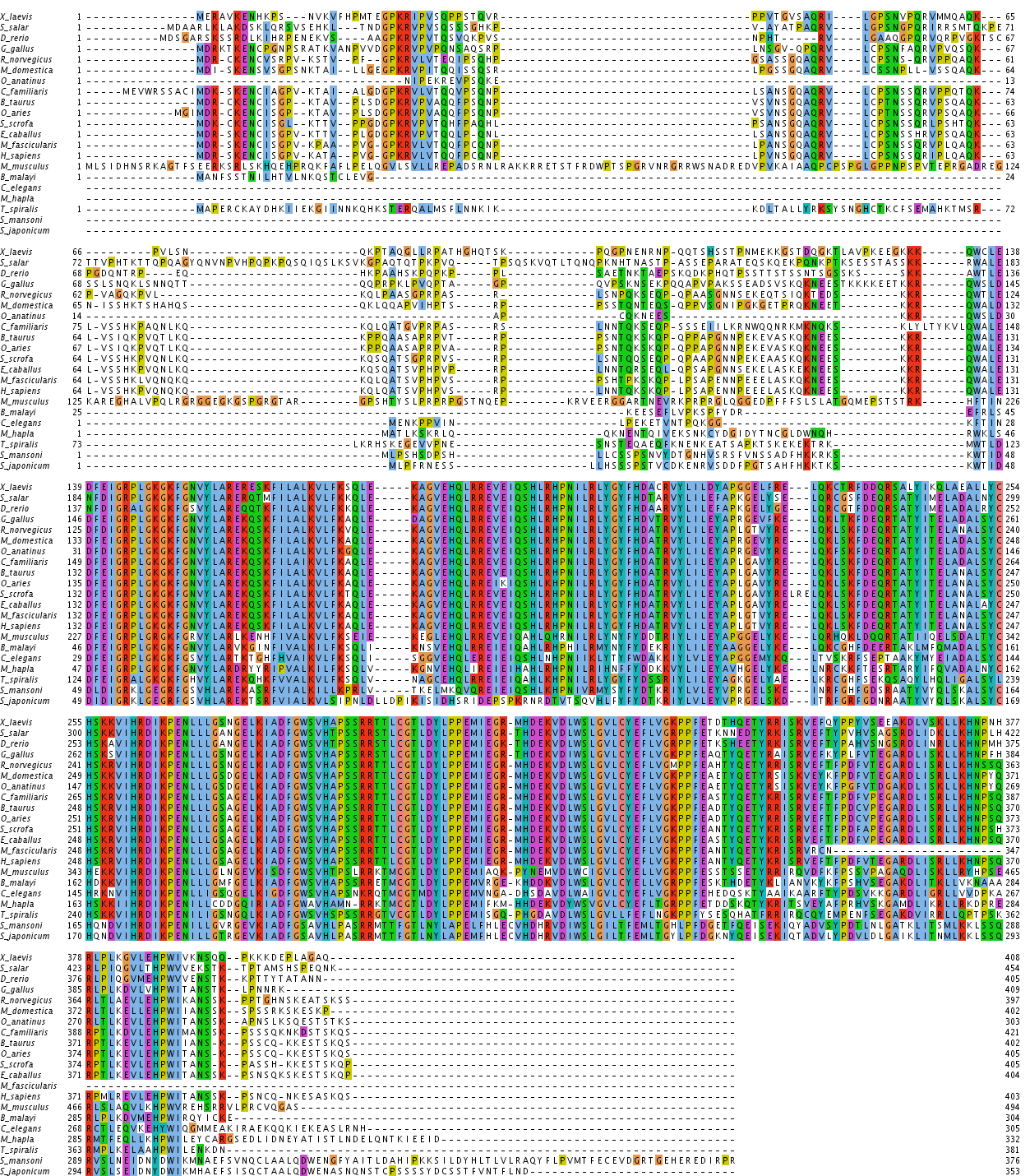

Supplement: Figure S1 — Sequence alignment of O01427. The indels are noted with red boxes. (DOC) [file pone.0018381.s004.doc]

**Figure S2.**


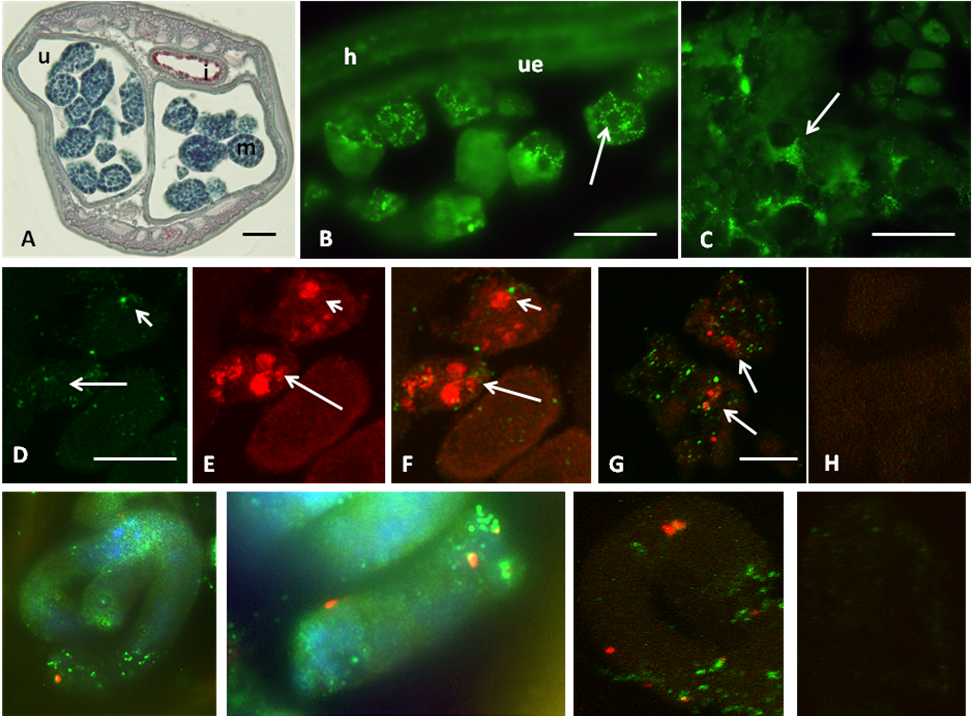

Supplement: Figure S2 — A. H&E stain of a midbody section of a female B. malayi showing the anatomy of the examined parasite sections. I, intestine; uterus, u; m, morula stage embryos. B. Granular staining (arrows) for Q19126 [XP_00189449.1] mRNA in the cytoplasm of morula stage embryos in the midbody region of a female B. malayi. Weeker staining was observed in the hypodermis (h) and the uterus epithelium (ue). The biotin labeled probe was detected using AlexaFluor 488-labeled streptavidin (green). C. Granular staining (arrows) for O01427 [XP_001892118.1] mRNA in the cytoplasm of egg cells and early morula stage embryos. The biotin labeled probe (label switch) was detected using AlexaFluor 488-labeled streptavidin (green). D-F. Confocal laser scanning microscopy (LSM). D Granular staining (arrows) for Q19126 [XP_00189449.1] mRNA in the cytoplasm of morulae. The biotin labeled probe was detected using AlexaFluor 488-labeled streptavidin (green). E. Identical section as in D showing granular staining (arrows) for O01427 [XP_001892118.1] mRNA in the same embyos. The digoxygenin labeled probe was detected using a Rhodamin conjugated anti-digoxygenin antibody (red). F. Overlay of D and E showing co-localization of expression of both genes. For 3 dimensional rotation of this section see Video S1. G Another overlay showing co-localization in morula stage embryos. H. Serial section to E showing the overlay for both sense probes (no DAPI) indicating the absence of specific labeling. I. Co-localization of RNA granules (arrow) positive for P46822 [XP_001895440.1] and Q17581 [XP_001895440.1] in pretzel stage embryos. J Pretzel stage embryo at higher magnification showing co-localization (arrows) in a number of granules. K. LSM image of co-localization in pretzel stage embryos showing the same pattern (no DAPI). L. Serial section to K, but hybridized with both sense probes (no DAPI) indicating the absence of specific labeling. Scale bar 10 µm. (DOC) [file pone.0018381.s005.doc]

**Figure S3.**

**
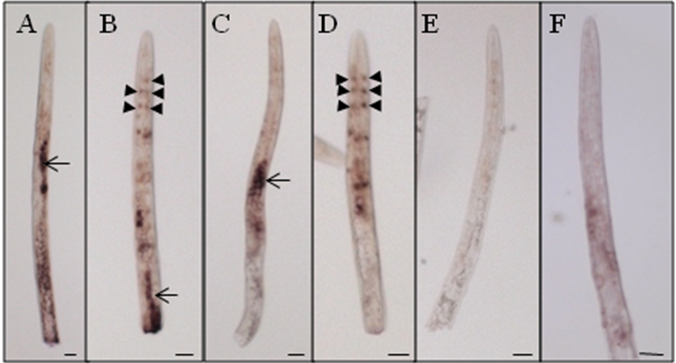
**

Supplement: Figure S3 — In situ hybridization of Q03601 (Minc18824) and Q20329 (Minc03587 and Minc058765) orthologs on Meloidogyne incognita L2. Transcripts were detected using immunostaining of digoxidenin-labeled antisense probes specific to Minc18824 (A,B) or specific to both Minc03587 and Minc058765 (C,D). For control, in situ hybridizations were performed with the sense Minc18824 (E) and sense Minc03587 -Minc058765 (F) probes. Expression co-localization was evidenced by the presence of the transcripts in the anterior part of the intestine (arrows) and the pharynx (arrow heads). Bar = 10 mm. (DOC) [file pone.0018381.s006.doc]

**Figure S4.**

A.


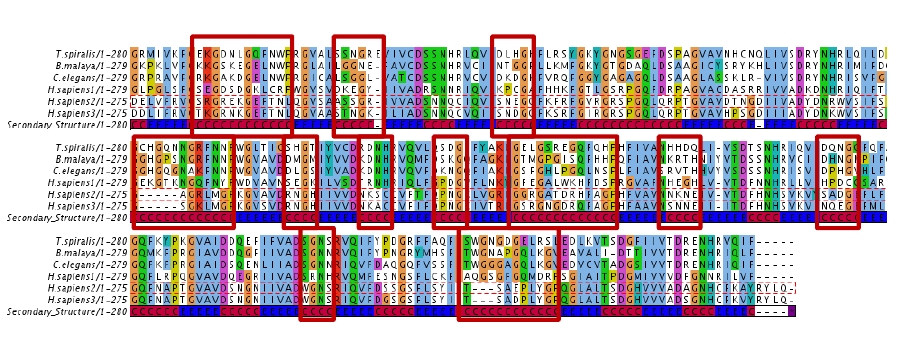


B.


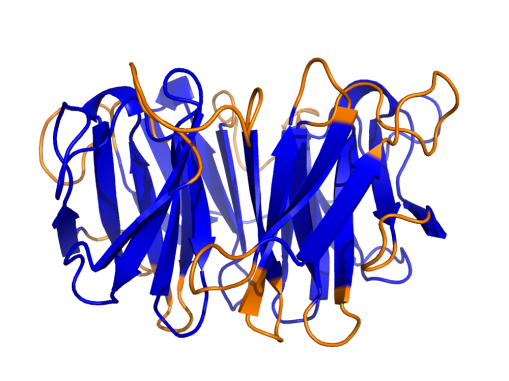

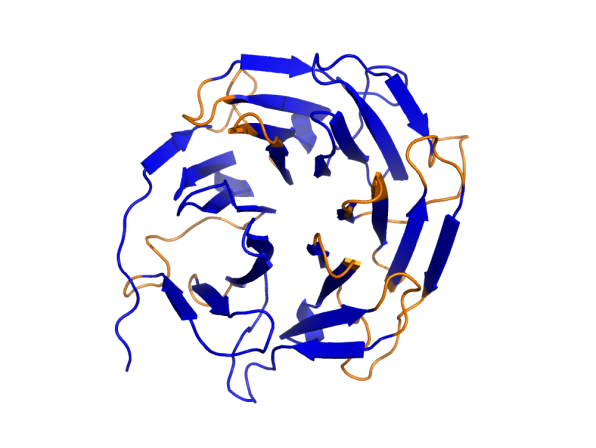

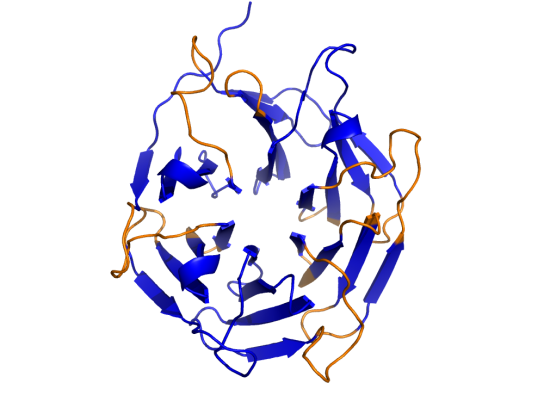


Bottom

Top

Side

Supplement: Figure S4 — A. Sequence alignment of Q03601 sequences from orthomcl, as well as homologous proteins from H. sapiens. The secondary structure prediction from the meta server is also shown in the alignment. Blue “E’s” represent beta-sheets, and red “C’s” represent loop regions. Much of the sequence diversity is isolated to the loop regions, thereby creating an accessible method for targeting Q03601 for a nematode specific drug. The loop regions are boxed in red. B. The boxed structures with sequence diversity are mapped to a homology model of Q03601 and highlighted in orange. (DOC) [file pone.0018381.s007.doc]

**Figure S5**.

A.


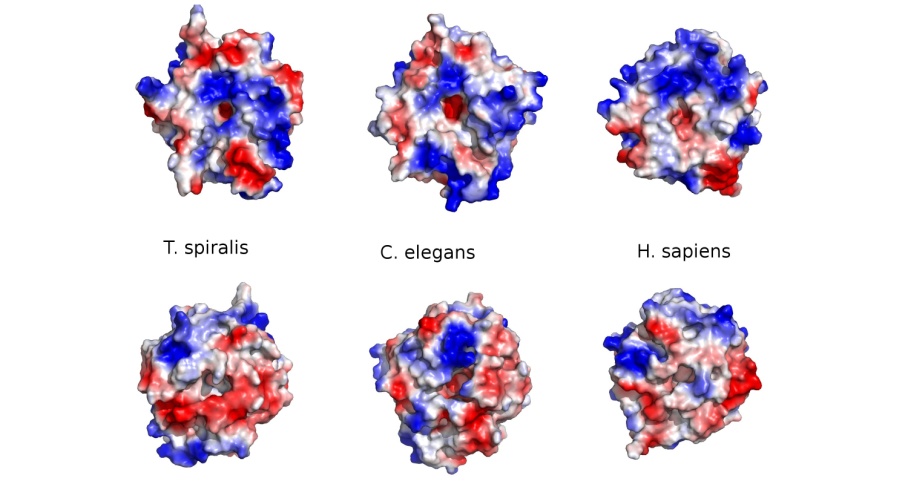


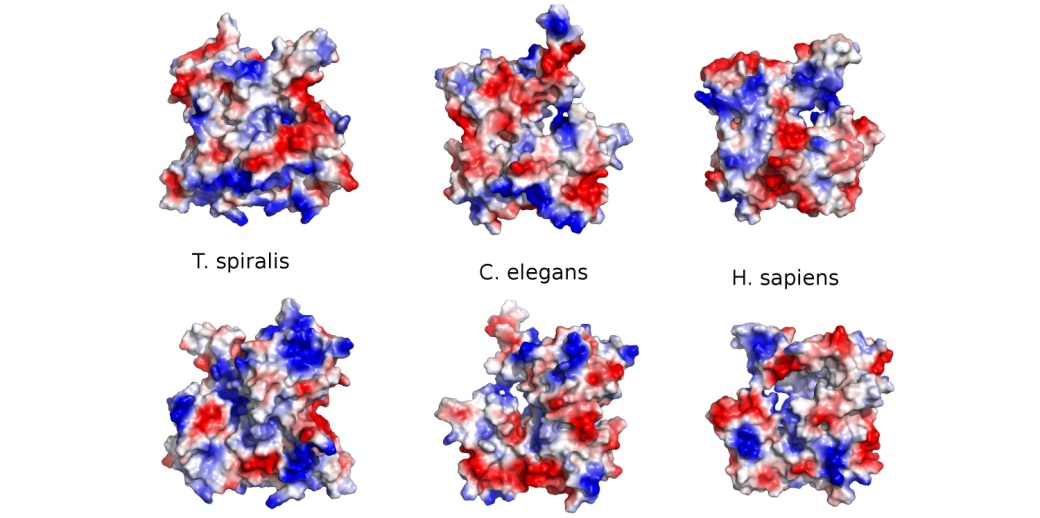

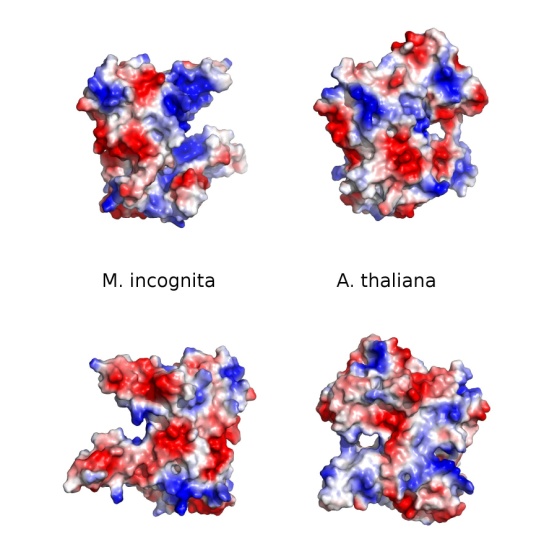


B.

C.

Supplement: Figure S5 — T. spiralis, C. elegans, and H. sapiens homology models of A. Q03601, B. Q20329, and C. M. hapla and A. thaliana homology models of Q20329 colored by electrostatic potential in vacuum. Q03601 did not have any protein sequences in A. thaliana with homology to M. hapla and M. incognita. Although regions of these proteins have homology to H. sapiens (A and B) and A. thaliana (C), the charges on the surface of H. sapiens and A. thaliana proteins are different from the charges on the surface of the nematode proteins. Further, orthomcl did not group the H. sapiens or A. thaliana proteins in the same orthologous groups as the nematode proteins. (DOC) [file pone.0018381.s008.doc]
