# Supplementary material for: Targeting Protein-Protein Interactions for Parasite Control
Source: PLoS One. 2011 Apr 27;6(4):e18381. doi: 10.1371/journal.pone.0018381 (PMC3083401; doi:10.1371/journal.pone.0018381)
Supplement: Table S1 — Full list of PPI targets in each of the three major groups: specific to nematodes (PPI-Nem), where both proteins contain indels with respect to human host (PPI-Indel2), with one indel with respect to human host (PPI-Indel1). (DOC) [file pone.0018381.s009.doc]

| **PPI** | | **Score** | | **RNAi Pheno^a^** | | **PDB Homo.** | | **Frac. of Len** | | **PPI Group^b^** | | **Function** | | **Stage^c^**  **Localization^d^** | |
| --- | --- | --- | --- | --- | --- | --- | --- | --- | --- | --- | --- | --- | --- | --- | --- |
| PPI-Nem | |  | |  | |  | |  | |  | |  | |  | |
|  | **Q03601/**  **Q20329** | | **253.7** | | **21/**  **315** | | **31.3/**  **30** | | **0.81/**  **0.65** | | **Nem**  **I** | | **ZnFinger, NHL repeat/**  **actin-like** | | **L1,Em,A / L1,L4,Eg,Em,A**  **PMR / ---** |
|  | O45666*/  O45666* | | 237.8 | | 32156/  32156 | | 35/  35 | | 0.53/  0.53 | | Nem  IM | | NHR-Znfinger/  NHR-Znfinger | | L1,L2,L4,Em,A / L1,L2,L4,Em,A  --- / --- |
|  | O45666*/  Q09528* | | 156.9 | | 32156/  No | | 35/  25.9 | | 0.53/  0.50 | | Nem  IM | | NHR-Znfinger/  NHR-Znfinger | | L1,L2,L4,Em,A / L,A  --- / --- |
|  | Q21234*/  Q21234* | | 150.0 | | 215/  215 | | No | | No | | Nem  IM | | Integrase | | L1,L2,L3,L4,Em,A/L1,L2,L3,L4,Em,A  --- / --- |
|  | Q8MYQ1/  Q22631 | | 147.4 | | 7/  No | | 39.6/  30.1 | | 0.96/  0.54 | | Nem  IM | | Ser-kinase/  thrombospondin | | L1 / L1,L2,L4,Em  --- / --- |
|  | O01489/  O01489 | | 135.0 | | 2/  2 | | No | | No | | Nem  IM | | ZnF protein | | L1,L2,L3,L4,Em,A / L1,L2,L3,L4,Em,A  PIMRNH / PIMRNH |
|  | Q03601/  O16266 | | 75.0 | | 21/  No | | No | | No | | Nem  I | | ZnFinger, NHL repeat/ | | L1,Em,A / Em  PMR / --- |
|  | Q9NDH1/  Q93413 | | 67.5 | | No/  254 | | No | | No | | Nem  M | | RNA-dep-RNA-pol/  DNA-RNA helicase | | L1,L2,L3,L4,Em,A / L1,L2,L3,L4,Em,A  --- / S |
| PPI-Indel2 | |  | |  | |  | |  | |  | |  | |  | |
|  | Q93716^+^/  Q93716^+^ | | 449.6 | | 321576/  321576 | | 100/  100 | | 0.99/  0.99 | | Indel2  IM | | NUDIX hydrolase domain | | L1,Em,A / L1,Em,A  PMH / PMH |
|  | P91988^+^/  P91988^+^ | | 449.5 | | 315/  315 | | 100/  100 | | 0.99/  0.99 | | Indel2  I | | Flavoprotein | | L1,Em,A / L1,Em,A  --- / --- |
|  | Q20471^+^/  Q20471^+^ | | 447.3 | | 321546/  321546 | | 100/  100 | | 0.97/  0.97 | | Indel2  IM | | Protein kinase | | L1,L4,Em,A / L1,L4,Em,A  --- / --- |
|  | P91851^+^/  P91851^+^ | | 434.6 | | 2/  2 | | 100/  100 | | 0.99/  0.99 | | Indel2  I | | nicotinate-nucleotide adenylyltransferase | | Em,A / Em,A  --- / --- |
|  | O18209^+^*/  Q17796^+^ | | 407.3 | | 321/  321546 | | 28/  41.5 | | 0.51/  0.95 | | Indel2  IM | | Protein Kinase/  Zinc finger | | L1,L4,Em,A / L1,L4,Em,A  E / EHIPR |
|  | P46822^+^/  P46822^+^ | | 396.3 | | 2/  2 | | 78.6/  78.6 | | 0.83/  0.83 | | Indel2  IM | | Tetratricopeptide | | L1,L4,Eg,Em,A / L1,L4,Eg,Em,A  --- / --- |
|  | **P46822^+^/**  **Q17581^+^** | | **392.7** | | **2/**  **32154** | | **78.6/**  **50.8** | | **0.83/**  **0.88** | | **Indel2**  **IM** | | **Tetratricopeptide/**  **Bromodomain** | | **L1,L4,Eg,Em,A/L1,Em,A**  **--- / ---** |
|  | O62305^+^*/  O62305^+^* | | 391.4 | | No/  No | | 100/  100 | | 0.91/  0.91 | | Indel2  I | | Protein kinase | | L1,L4,Em,A / L1,L4,Em,A  --- / --- |
|  | Q7JP75^+^/  Q19749^+^ | | 381.0 | | 3215746/  325 | | 49.7/  61.6 | | 0.73/  0.93 | | Indel2  IM | | Actin Binding/  Acyltransferase | | L1,Eg,Em,A / L1,L2,L4,Em,A  --- / PIMR |
|  | O16299^+^/  O16299^+^ | | 377.4 | | 3/3 | | 65/  65 | | 0.98/  0.98 | | Indel2  M | | MCM protein 7 | | L1,L2,L4,Em,A / L1,L2,L4,Em,A  --- / --- |
| **PPI-Indel1** | |  | |  | |  | |  | |  | |  | |  | |
|  | P34475^+^*/  Q19207* | | 475.9 | | 3217/  321546 | | 99.5/  99.7 | | 0.99/  0.53 | | Indel1  IM | | Tubulin/ Hydroxymethylglutaryl-CoA reductase | | L1,L4,Em,A / L1,L2,Eg,Em,A  RE / --- |
|  | ***O01427^+^*/***  ***Q19126*** | | ***434.5*** | | ***32174/***  ***3215*** | | ***100/***  ***86.4*** | | ***0.99/***  ***0.83*** | | ***Indel1***  ***IM*** | | ***Protein kinase/***  ***ATPase*** | | ***L1,L4,Em,A / L1,L4,Eg,Em,A***  ***RE / whole body*** |
|  | P39745/  Q9BIB3^+^* | | 426.9 | | 32157/  3215 | | 100/  100 | | 0.99/  0.54 | | Indel1  IM | | Protein kinase/  Lipase | | L1,L2,L4,Em,A / L1,A  --- / --- |
|  | **Q95005/**  **Q19207^+^*** | | **426.0** | | **32156/**  **321546** | | **100/**  **99.7** | | **0.99/**  **0.53** | | **Indel1**  **IM** | | **Proteasome/Hydroxymethylglutaryl-CoA reductase** | | **L1,L4,Em,A / L1,L2,Eg,Em,A**  **PM / ---** |
|  | Q19207^+^*/  Q22799 | | 425.7 | | 3215746/  321546 | | 99.7/  100 | | 0.53/  0.98 | | Indel1  IM | | Hydroxymethylglutaryl-CoA reductase/Dynein light chain | | L1,L2,Eg,Em,A / A  --- / PNI |
|  | P39745*/  O62305^+^* | | 420.6 | | 32157/  No | | 100/  100 | | 0.99/  0.91 | | Indel1  IM | | Protein kinase like /  Protein kinase | | L1,L2,L4,Em,A / L1,L4,Em,A  --- / --- |
|  | P39745^*^/  O16299^+^ | | 413.6 | | 32157/3 | | 100/  65 | | 0.99/  0.98 | | Indel1  IM | | Protein kinase – like /  MCM protein 7 | | L1,L4,Em,A / L1,L2,L4,Em,A  --- / --- |
|  | P34442^+^*/  Q27488 | | 406.1 | | 21/32 | | 40.3/  100 | | 0.88/  0.99 | | Indel1  IM | | Protein-tyrosine phosphatase / Proteasome | | L1,L4,Em,A / L1,L4,EgEm,A  EP / --- |
|  | O17915/  P46769^+^ | | 399.6 | | 32156/  32157 | | 100/  100 | | 0.99/  0.99 | | Indel1  IM | | GTPase activity /  Ribosomal protein | | L1,L2,L3,L4,Em,A/L1,L2,L3,L4,Eg,Em,A  --- / PIMH |
|  | Q07750^+^/  P10986 | | 399.6 | | 31576/  32157 | | 100/  100 | | 0.99/  0.99 | | Indel1  I | | Actin binding /  Actin | | L1,L4,Em,A / L1,L2,L4,Eg,Em,A  --- / --- |
|  | O17915/  Q20206^+^ | | 399.5 | | 32156/  3215 | | 100/  100 | | 0.99/  0.99 | | Indel1  IM | | GTPase activity /  Ribosomal protein | | L1,L2,L3,L4,Em,A / L1,L2,L4,Eg,Em,A  --- / --- |
|  | Q22799/  Q93572^+^ | | 399.3 | | 3215746/  321 | | 100/  100 | | 0.98/  0.99 | | Indel1  IM | | Dynein light chain / Ribosomal protein | | A / L1,L2,L3,L4,Em,A  PNMIN / PIMN |

* indicates druggable, PPIs in bold italic were tested with *in situ* hybridization, and + indicates protein with indel, ^a^ RNAi phenotype 1=Larval/Adult Lethal/Arrest, 2=Embryonic Lethal, 3=Sterility, 4=Morphology, 5=Growth, 6=Movement, 7=Vulva, 8=Other; ^b^ Indicates analysis group (Nem, Indel2, and Indel1) and also the database where the PPI was found (M=MINT and I=IntAct), ^c^ Stages are listed as L1, L2, L3, L4, egg (Eg), embryo (Em), and Adult (A), ^d^ Localization in *C. elegans* listed as pharynx (P), intestine (I), reproductive (R), muscle (M), hypodermis (H), nervous system (N), somatic (S), embryo (E)
