# Supplementary material for: Targeting Protein-Protein Interactions for Parasite Control
Source: PLoS One. 2011 Apr 27;6(4):e18381. doi: 10.1371/journal.pone.0018381 (PMC3083401; doi:10.1371/journal.pone.0018381)
Supplement: Table S2 — PPI-Indel1: Plant parasite PPIs with one indel with respect to Arabidopsis host. The cutoff score was 399. The following symbols were used to indicate specific features: * indicates druggable, PPIs with + indicate protein with indel, a RNAi phenotype 1 = Larval/Adult Lethal/Arrest, 2 = Embryonic Lethal, 3 = Sterility, 4 = Morphology, 5 = Growth, 6 = Movement, 7 = Vulva, 8 = Other; b Indicates analysis group (Nem, Indel2, and Indel1) and also the database where the PPI was found (M = MINT and I = IntAct), c Stages are listed as L1, L2, L3, L4, egg (Eg), embryo (Em), and Adult (A), d Localization in C. elegans listed as pharynx (P), intestine (I), reproductive (R), muscle (M), hypodermis (H), nervous system (N), somatic (S), embryo (E). (DOC) [file pone.0018381.s010.doc]

| **PPI** | **Score** | **RNAi Pheno** | **PDB Homo** | **Frac. of Len** | **PPI Group^b^** | **Function** | **Stage^c^**  **Localization^d^** |
| --- | --- | --- | --- | --- | --- | --- | --- |
| P39745*/  Q86G90^+^ | 442.1 | 32157/  32574 | 100/  100 | 0.99/  0.99 | Indel1  IM | Protein Kinase like / Ran Binding Protein 1 | L1,L2,L4,Em,A / L1,L2,L4,Em,A  --- / --- |
| P91302*/  Q86G90^+^ | 441.6 | 3156/  32574 | 100/  100 | 0.98/  0.99 | Indel1  I | Ubiquitin supergroup / Ran Binding Protein 1 | L4,A / L1,L2,L4,Em,A  Most tissue / --- |
| Q95005/  Q19207*^+^ | 426.1 | 32156/  321546 | 100/  99.7 | 0.99/  0.53 | Indel1  I | Proteasome/Hydroxymethylglutaryl-CoA reductase | L1,L4,Em,A / L1,L2,L4,Eg,Em,A  PM / --- |
| P39745*/  O62305*^+^ | 421.6 | 32157/  No | 100/  100 | 0.99/  0.96 | Indel1  IM | Protein kinase like / Protein kinase | L1,L2,L4,Em,A / L1,L2,L4,Em,A  --- / --- |
| Q19546^+^/  O01427* | 418.5 | 1574/ 32174 | 48.2/  100 | 0.89/  0.99 | Indel1  IM | DEAD-like helicase / serine-threonine kinase activity | L1,L2,L4,Em,A / L1,L4,Em,A  EHI / RE |
| O45605*^+^/  P35129 | 402.1 | 2/  3215746 | 34/  100 | 0.86/  0.99 | Indel1  M | Cytochrome P450 / Ubiquitin-conjugating enzyme, E2 | A / L1,Em,A  --- / ENPHM |
| O45605*^+^/  P50880 | 401.2 | 2/  3215 | 34/  98 | 0.86/  0.99 | Indel1  M | Cytochrome P450 / Ribosomal protein | A / L1,L2,L4,Eg,Em,A  --- / --- |
| O17915/  P46769^+^ | 399.6 | 32156/  32157 | 100/  100 | 0.99/  0.99 | Indel1  IM | GTPase / Ribosomal Protein | L1,L2,L3,L4,Eg,Em,A / L1,L2,L3,L4,Eg,Em,A  --- / PIMHN |
| Q07750^+^/  P10986 | 399.6 | 31576/  32157 | 100/  100 | 0.99/  0.99 | Indel1  I | Actin binding / Actin | L1,L4,Em,A / L1,L2,L4,Eg,Em,A  --- / --- |
| Q95008^+^/  Q95005 | 399.2 | 3216/  32156 | 99.1/  100 | 0.99/  0.99 | Indel1  IM | Proteosome / Proteosome | L1,L4,Eg,Em,A / L1,L4,Em,A  --- / PM |
