# Supplementary material for: Targeting Protein-Protein Interactions for Parasite Control
Source: PLoS One. 2011 Apr 27;6(4):e18381. doi: 10.1371/journal.pone.0018381 (PMC3083401; doi:10.1371/journal.pone.0018381)
Supplement: Table S3 — PPI-Indel2: Plant parasite PPIs where both proteins contain indels with respect to Arabidopsis host. The cutoff score was 393. The following symbols were used to indicate specific features: * indicates druggable, PPIs with + indicate protein with indel, a RNAi phenotype 1 = Larval/Adult Lethal/Arrest, 2 = Embryonic Lethal, 3 = Sterility, 4 = Morphology, 5 = Growth, 6 = Movement, 7 = Vulva, 8 = Other; b Indicates analysis group (Nem, Indel2, and Indel1) and also the database where the PPI was found (M = MINT and I = IntAct), c Stages are listed as L1, L2, L3, L4, egg (Eg), embryo (Em), and Adult (A), d Localization in C. elegans listed as pharynx (P), intestine (I), reproductive (R), muscle (M), hypodermis (H), nervous system (N), somatic (S), embryo (E). (DOC) [file pone.0018381.s011.doc]

| **PPI** | **Score** | **RNAi Pheno** | **PDB Homo.** | **Frac of Len.** | **PPI Group^b^** | **Function** | **Stage^c^**  **Localization^d^** |
| --- | --- | --- | --- | --- | --- | --- | --- |
| P42170**^+^**/  P42170**^+^** | 449.0 | 321574/  321574 | 99.6/  99.6 | 0.99/  0.99 | Indel2  IM | Ribonucleotide reductase | L1,L4,Em,A / L1,L4,Em,A  I / I |
| P46561**^+^**/  P46769**^+^** | 445.5 | 3215/  32157 | 98.7/  100 | 0.93/  0.99 | Indel2  I | ATPase / Ribosomal Protein | L1,L2,L4,Em,A / L1,L2,L3,L4,Eg,Em,A  PMRHNI / PIMHN |
| Q95008**^+^**/  Q9XXK1**^+^** | 445.4 | 3216/  3215 | 99.1/  98.6 | 0.99/  0.94 | Indel2  IM | Proteasome/ATPase | L1,L4,Eg,Em,A / L1,L2,L3,L4,Eg,Em,A  --- / --- |
| Q20483***^+^**/  P46769**^+^** | 438.4 | 546/  32157 | 33.7/  100 | 0.76/  0.99 | Indel2  IM | Serine-threonine protein kinase-related/Ribosomal Protein | L1,L4,Em,A / L1,L2,L3,L4,Eg,Em,A  PI / PIMH |
| P91851**^+^**/  P91851**^+^** | 434.6 | 2/  2 | 100/  100 | 0.99/  0.99 | Indel2  I | Probable nicotinate-nucleotide adenylyltransferase | L1,L4,Em,A / L1,L4,Em,A  --- / --- |
| Q86G90**^+^**/  Q86G90**^+^** | 434.5 | 32574/  32574 | 100/  100 | 0.99/  0.99 | Indel2  I | Ran Binding Protein | L1,L2,L4,Em,A / L1,L2,L4,Em,A  --- / --- |
| P52874**^+^**/  P34286**^+^** | 412.9 | 321574/  321746 | 30.2/  100 | 0.96/  0.99 | Indel2  IM | Pre-mRNA cleavage complex II Clp1 / Proteasome | L1,L2,L4,Eg,Em,A / L1,Eg,Em,A  --- / P |
| O17917**^+^**/  Q23670***^+^** | 410.2 | 32157/  321 | 47.6/  66 | 0.17/  0.90 | Indel2  M | Zinc Finger / DNA topoisomerase | L1,L4,Em,A / L1,L4,Eg,Em,A  --- / --- |
| Q09584**^+^**/  Q18409**^+^** | 408.6 | 3217/  321574 | 66.5/  100 | 0.91/  0.60 | Indel2  IM | RNA recognition motif, RNP-1 / RNA recognition motif, RNP-1 | L1,L4,Em,A / L1,L2,L4,Em,A  --- / --- |
| Q21029***^+^**/  Q9XXK1**^+^** | 393.3 | No/  3215 | 61.3/  98.6 | 0.83/  0.94 | Indel2  M | Serine/threonine protein kinase / ATPase | L1,L2,L4,Em,A / L1,L2,L3,L4,Eg,Em,A  IH / --- |
