# Supplementary material for: Targeting Protein-Protein Interactions for Parasite Control
Source: PLoS One. 2011 Apr 27;6(4):e18381. doi: 10.1371/journal.pone.0018381 (PMC3083401; doi:10.1371/journal.pone.0018381)
Supplement: Table S4 — The total score broken down into terms for the full list of PPI targets in each of the three major groups: specific to nematodes (PPI-Nem), where both proteins contain indels with respect to human host (PPI-Indel2), with one indel with respect to human host (PPI-Indel1). The score is broken down into the terms in the scoring function, so that the PPI can be evaluated for further investigation. (DOC) [file pone.0018381.s012.doc]

| **PPI** | | **Score** | | **RNAi**  **(150)** | | **PDB**  **(200)** | | **Druggability**  **(100)** | | **Indel**  **(100)** | | | |
| --- | --- | --- | --- | --- | --- | --- | --- | --- | --- | --- | --- | --- | --- |
| PPI-Nem | |  | |  | |  | |  | |  | |  | |
|  | **Q03601/**  **Q20329** | | **253.7** | | **150** | | **103.7** | | -- | | **--** | |  |
|  | O45666*/  O45666* | | 237.8 | | 150 | | 87.8 | | -- | | -- | |  |
|  | O45666*/  Q09528* | | 156.9 | | 75 | | 81.9 | | -- | | -- | |  |
|  | Q21234*/  Q21234* | | 150.0 | | 150 | | No | | -- | | -- | |  |
|  | Q8MYQ1/  Q22631 | | 147.4 | | 37.5 | | 109.9 | | -- | | -- | |  |
|  | O01489/  O01489 | | 135.0 | | 135 | | No | | -- | | -- | |  |
|  | Q03601/  O16266 | | 75.0 | | 75 | | No | | -- | | -- | |  |
|  | Q9NDH1/  Q93413 | | 67.5 | | 67.5 | | No | | -- | | -- | |  |
| PPI-Indel2 | |  | |  | |  | |  | |  | |  | |
|  | Q93716^+^/  Q93716^+^ | | 449.6 | | 150 | | 199.6 | | 0 | | 100 | |  |
|  | P91988^+^/  P91988^+^ | | 449.5 | | 150 | | 199.5 | | 0 | | 100 | |  |
|  | Q20471^+^/  Q20471^+^ | | 447.3 | | 150 | | 197.3 | | 0 | | 100 | |  |
|  | P91851^+^/  P91851^+^ | | 434.6 | | 135 | | 199.6 | | 0 | | 100 | |  |
|  | O18209^+^*/  Q17796^+^ | | 407.3 | | 150 | | 107.3 | | 50 | | 100 | |  |
|  | P46822^+^/  P46822^+^ | | 396.3 | | 135 | | 161.3 | | 0 | | 100 | |  |
|  | **P46822^+^/**  **Q17581^+^** | | **392.7** | | **142.5** | | **150.2** | | **0** | | **100** | |  |
|  | O62305^+^*/  O62305^+^* | | 391.4 | | 0 | | 191.4 | | 100 | | 100 | |  |
|  | Q7JP75^+^/  Q19749^+^ | | 381.0 | | 142.5 | | 138.5 | | 0 | | 100 | |  |
|  | O16299^+^/  O16299^+^ | | 377.4 | | 120 | | 157.4 | | 0 | | 100 | |  |
| **PPI-Indel1** | |  | |  | |  | |  | |  | |  | |
|  | P34475^+^*/  Q19207* | | 475.9 | | 150 | | 175.9 | | 100 | | 50 | |  |
|  | ***O01427^+^*/***  ***Q19126*** | | ***434.5*** | | ***150*** | | ***184.5*** | | ***50*** | | 50 | |  |
|  | P39745/  Q9BIB3^+^* | | 426.9 | | 150 | | 176.9 | | 50 | | 50 | |  |
|  | **Q95005/**  **Q19207^+^*** | | **426.0** | | **150** | | **176.0** | | **50** | | 50 | |  |
|  | Q19207^+^*/  Q22799 | | 425.7 | | 150 | | 175.7 | | 50 | | 50 | |  |
|  | P39745*/  O62305^+^* | | 420.6 | | 75 | | 195.6 | | 100 | | 50 | |  |
|  | P39745*/  O16299^+^ | | 413.6 | | 135 | | 178.6 | | 50 | | 50 | |  |
|  | P34442^+^*/  Q27488 | | 406.1 | | 142.5 | | 163.6 | | 50 | | 50 | |  |
|  | O17915/  P46769^+^ | | 399.6 | | 150 | | 199.6 | | 0 | | 50 | |  |
|  | Q07750^+^/  P10986 | | 399.6 | | 150 | | 199.6 | | 0 | | 50 | |  |
|  | O17915/  Q20206^+^ | | 399.5 | | 150 | | 199.5 | | 0 | | 50 | |  |
|  | Q22799/  Q93572^+^ | | 399.3 | | 150 | | 199.3 | | 0 | | 50 | |  |

* indicates druggable, PPIs in bold italic were tested with *in situ* hybridization, and + indicates protein with indel, ^a^ RNAi phenotype 1=Larval/Adult Lethal/Arrest, 2=Embryonic Lethal, 3=Sterility, 4=Morphology, 5=Growth, 6=Movement, 7=Vulva, 8=Other; ^b^ Indicates analysis group (Nem, Indel2, and Indel1) and also the database where the PPI was found (M=MINT and I=IntAct), ^c^ Stages are listed as L1, L2, L3, L4, egg (Eg), embryo (Em), and Adult (A), ^d^ Localization in *C. elegans* listed as pharynx (P), intestine (I), reproductive (R), muscle (M), hypodermis (H), nervous system (N), somatic (S), embryo (E)
