# Supplementary material for: Targeting Protein-Protein Interactions for Parasite Control
Source: PLoS One. 2011 Apr 27;6(4):e18381. doi: 10.1371/journal.pone.0018381 (PMC3083401; doi:10.1371/journal.pone.0018381)
Supplement: Table S5 — Oligonucleotide probes used for ISH. Sense probes with the dogoxigenin label were used as controls for each probe. (DOC) [file pone.0018381.s013.doc]

| Gene name | orientation | label | Sequence (5’-3’) |
| --- | --- | --- | --- |
| Q03601 | Sense | Minc18824F | TCA AGG GAG TGA AGA TGA TGC |
| Q03601 | Anti-sense | Minc18824R | CAC CTT TAA ATT GCC CTG GA |
| Q20329 | Sense | Minc05876F2 | AAA GAT GGG CGT TTT CAA TG |
| Q20329 | Anti-sense | Minc05876R2 | TGA GGG CAC CCG AAC TAA TA |
