# Supplementary material for: Targeting Protein-Protein Interactions for Parasite Control
Source: PLoS One. 2011 Apr 27;6(4):e18381. doi: 10.1371/journal.pone.0018381 (PMC3083401; doi:10.1371/journal.pone.0018381)
Supplement: Table S6 — Taxonomically restricted orthologous groups. (DOC) [file pone.0018381.s014.doc]

|  | **with Human** | **Ex Human** | **with Arab** | **Ex Arab** | **Ex Arab & H** |
| --- | --- | --- | --- | --- | --- |
| **HPN+HPF+PPN (Bin 1/2)** | 1808 | 31 |  |  |  |
| **HPN+HPF+PPN+FLN**  **(Bin 3/4)** | 1779 | 31 |  |  |  |
| **HPF**  **(Bin 5/6)** | 4216 | 3001 |  |  |  |
| **HPF+FLN**  **(Bin 7 & 8)** | 3347 | 119 |  |  |  |
| **PPN**  **(Bin 9/10/11)** | --- | --- | 1858 | 4433 | 3202 |
| **PPN+FLN**  **(Bin 12/13/14)** | --- | --- | 1801 | 2284 | 1112 |
| **HPN+PPN**  **(Bin 15/16)** | 2172 | 267 |  |  |  |
| **HPN+PPN+FLN**  **(Bin 17/18)** | 2132 | 255 |  |  |  |
| **HPN**  **(Bin 19/20)** | 3210 | 481 |  |  |  |
| **HPN+FLN**  **(Bin 21/22)** | 3091 | 419 |  |  |  |
