# Supplementary material for: Targeting Protein-Protein Interactions for Parasite Control
Source: PLoS One. 2011 Apr 27;6(4):e18381. doi: 10.1371/journal.pone.0018381 (PMC3083401; doi:10.1371/journal.pone.0018381)
Supplement: Table S7 — PPIs from MINT and IntAct (PPI-Nem). (DOC) [file pone.0018381.s015.doc]

|  | **MINT** | | **IntAct** | |
| --- | --- | --- | --- | --- |
|  | **Ex Hs** | **Ex Ar & Hs** | **Ex Hs** | **Ex Ar & Hs** |
| **HPN+HPF+PPN+FLN**  **(Bin 4)** | 0 |  | 0 |  |
| **HPF+FLN**  **(Bin 8)** | 0 |  | 0 |  |
| **HPN+PPN+FLN**  **(Bin 18)** | 1 |  | 3 |  |
| **HPN+FLN**  **(Bin 22)** | 3 |  | 5 |  |
| **PPN+FLN**  **(Bin 14)** |  | 7 |  | 15 |
