# Supplementary material for: Targeting Protein-Protein Interactions for Parasite Control
Source: PLoS One. 2011 Apr 27;6(4):e18381. doi: 10.1371/journal.pone.0018381 (PMC3083401; doi:10.1371/journal.pone.0018381)
Supplement: Table S8 — PPIs from MINT and IntAct (PPI-Indel). (DOC) [file pone.0018381.s016.doc]

|  | **Overlap** | **MINT** | **IntAct** |
| --- | --- | --- | --- |
| **HPN+HPF+PPN+FLN**  **(Bin 3)** | 165 | 190 | 238 |
| **HPF+FLN**  **(Bin 7)** | 412 | 464 | 597 |
| **HPN+PPN+FLN**  **(Bin 15)** | 191 | 220 | 277 |
| **HPN+FLN**  **(Bin 21)** | 388 | 436 | 571 |
| **PPN+FLN**  **(Bin 9)** | 161 | 185 | 219 |
