# Supplementary material for: Targeting Protein-Protein Interactions for Parasite Control
Source: PLoS One. 2011 Apr 27;6(4):e18381. doi: 10.1371/journal.pone.0018381 (PMC3083401; doi:10.1371/journal.pone.0018381)
Supplement: Table S9 — PPI-Nem: Unique protein-protein interactions in each bin found from the MINT and IntAct Databases. Interactions in bold were found in both the MINT and IntAct Databases. (DOC) [file pone.0018381.s017.doc]

| **Bins** | **Database** | **PPI Interactions** |
| --- | --- | --- |
| **HPN+FLN**  **(Bin 22)** | MINT | **Q21234/Q21234**, **Q8MYQ1/Q22631**, Q9NDH1/Q93431 |
| **HPN+FLN**  **(Bin 22)** | IntAct | **Q8MYQ1/Q22631**, **Q21234/Q21234**, Q03601/O16266, Q03601/Q20329, Q18341/Q18341 |
| **PPN+FLN ex Hs**  **(Bin 14)** | MINT | **O01489/O01489**, **O45666/O45666**, **P34371/P34371**, **Q09528/O45666**, **Q09528/Q21372**,**Q18869/P34383**,**Q9XTG8/Q8I4J6** |
| **PPN+FLN ex Hs**  **(Bin 14)** | IntAct | **O01489/O01489**, **P34371/P34371**, **O45666/O45666**, Q966L9/Q966L9, O01836/O01836, Q18341/Q18341, Q9XV46/Q9XV46, **Q18869/P34383**, **Q9XTG8/Q8I4J6**, **Q09528/Q21372**, **O45666/Q09528**, Q03601/O16266, Q03601/Q20329, Q19016/O44490, Q19016/Q9N5S5 |
| **HPN+PPN+FLN**  **(Bin 18)** | MINT | Q9NDH1/Q93431 |
| **HPN+PPN+FLN**  **(Bin 18)** | IntAct | Q03601/O16266, Q03601/Q20329, Q18341/Q18341 |
