# Supplementary material for: Targeting Protein-Protein Interactions for Parasite Control
Source: PLoS One. 2011 Apr 27;6(4):e18381. doi: 10.1371/journal.pone.0018381 (PMC3083401; doi:10.1371/journal.pone.0018381)
Supplement: Table S10 — PPI-Nem: Unique protein-protein interactions in each bin found from the MINT and IntAct Databases where both proteins involved in the protein-protein interaction had an RNAi phenotype. Interactions in bold were found in both the MINT and IntAct Databases. (DOC) [file pone.0018381.s018.doc]

| **Bins** | **Database** | **PPI Interaction (RNAi Score)** |
| --- | --- | --- |
| **HPN+FLN**  **(Bin 22)** | MINT | **Q21234/Q21234 (150)** |
| **HPN+FLN**  **(Bin 22)** | IntAct | **Q21234/Q21234 (150)**, Q03601/Q20329 (150) |
| **PPN+FLN ex Hs**  **(Bin 14)** | MINT | **O01489/O01489** (135), **O45666/O45666** (150) |
| **PPN+FLN ex Hs**  **(Bin 14)** | IntAct | **O01489/O01489** (135), **O45666/O45666** (150), Q03601/Q20329 (150) |
| **HPN+PPN+FLN**  **(Bin 18)** | IntAct | Q03601/Q20329 (150) |
