# Supplementary material for: Targeting Protein-Protein Interactions for Parasite Control
Source: PLoS One. 2011 Apr 27;6(4):e18381. doi: 10.1371/journal.pone.0018381 (PMC3083401; doi:10.1371/journal.pone.0018381)
Supplement: Table S11 — PPI-Nem: PPIs in which one of the proteins has an RNAi phenotype. These proteins might be good targets for subsequent RNAi experiments. Interactions in bold were found in both the MINT and IntAct Databases. (DOC) [file pone.0018381.s019.doc]

| **Bins** | **Database** | **PPI Interaction (RNAi Score)** |
| --- | --- | --- |
| **HPN+FLN**  **(Bin 22)** | MINT | **Q8MYQ1/Q22631** (37.5), Q9NDH1/Q93431 (67.5) |
| **HPN+FLN**  **(Bin 22)** | IntAct | **QMYQ1/Q22631** (37.5) |
| **PPN+FLN ex Hs**  **(Bin 14)** | MINT | Q09528/O45666 (75) |
| **PPN+FLN ex Hs**  **(Bin 14)** | IntAct | Q03601/O16266 (75) |
| **HPN+PPN+FLN**  **(Bin 18)** | MINT | Q9NDH1/Q93431 (67.5) |
| **HPN+PPN+FLN**  **(Bin 18)** | IntAct | O45666/Q09528 (75), Q03601/O16266 (75), |
