# Supplementary material for: Targeting Protein-Protein Interactions for Parasite Control
Source: PLoS One. 2011 Apr 27;6(4):e18381. doi: 10.1371/journal.pone.0018381 (PMC3083401; doi:10.1371/journal.pone.0018381)
Supplement: Table S12 — Conversion of UniprotIDs to C. elegans gene IDs. (DOC) [file pone.0018381.s020.doc]

| **UniprotID** | |  | **C. elegans Gene** | |  | **UniProtID** | |  | **C. elegans Gene** | |
| --- | --- | --- | --- | --- | --- | --- | --- | --- | --- | --- |
| Q03601 | Q20329 |  | F54G8.4 | F42C5.9 |  | P34442 | Q27488 |  | F54C8.4 | D1054.2 |
| O45666 | O45666 |  | K10C3.6c | K10C3.6c |  | O17915 | P46769 |  | K01G5.4 | B0393.1 |
| O45666 | Q09528 |  | K10C3.6c | E02H1.7 |  | Q07750 | P10986 |  | C38C3.5b | M03F4.2a |
| Q21234 | Q21234 |  | K04G7.1 | K04G7.1 |  | O17915 | Q20206 |  | K01G5.4 | F40F11.1 |
| Q8MYQ1 | Q22631 |  | F31E3.2c | T21B6.3 |  | Q22799 | Q93572 |  | T26A5.9 | F25H2.10 |
| O01489 | O01489 |  | C13F10.7 | C13F10.7 |  | P42170 | P42170 |  | C03C10.3 | C03C10.3 |
| Q03601 | O16266 |  | F54G8.4 | F40A3.6 |  | P46561 | P46769 |  | C34E10.6 | B0393.1 |
| Q9NDH1 | Q93413 |  | F26A3.8 | D2005.5 |  | Q95008 | Q9XXK1 |  | F25H2.9 | H28O16.1a |
| Q93716 | Q93716 |  | F43G9.5 | F43G9.5 |  | Q20483 | P46769 |  | F46G11.3 | B0393.1 |
| P91988 | P91988 |  | F25H9.6 | F25H9.6 |  | P91851 | P91851 |  | F26H9.4 | F26H9.4 |
| Q20471 | Q20471 |  | F46F2.2c | F46F2.2c |  | Q86G90 | Q86G90 |  | F59A2.1b | F59A2.1b |
| P91851 | P91851 |  | F26H9.4 | F26H9.4 |  | P52874 | P34286 |  | F59A2.4a | C02F5.9 |
| O18209 | Q17796 |  | Y53C12A.1 | C07G1.5 |  | O17917 | Q23670 |  | K01G5.1 | K12D12.1 |
| P46822 | P46822 |  | C18C4.10b | C18C4.10b |  | Q09584 | Q18409 |  | K04G7.10 | C33H5.12a |
| P46822 | Q17581 |  | C18C4.10b | C01H6.7a |  | Q21029 | Q9XXK1 |  | F59A6.1 | H28O16.1a |
| O62305 | O62305 |  | K11E8.1c | K11E8.1c |  | P39745 | Q86G90 |  | F43C1.2b | F59A2.1b |
| Q7JP75 | Q19749 |  | F11H8.4b | F23B12.5 |  | P91302 | Q86G90 |  | F46F11.4 | F59A2.1b |
| O16299 | O16299 |  | F32D1.1 | F32D1.1 |  | Q95005 | Q19207 |  | C36B1.4 | F08F8.2 |
| P34475 | Q19207 |  | F58A4.8 | F08F8.2 |  | P39745 | O62305 |  | F43C1.2b | K11E8.1c |
| O01427 | Q19126 |  | B0207.4 | F02E8.1 |  | Q19546 | O01427 |  | F18C5.2 | B0207.4 |
| P39745 | Q9BIB3 |  | F43C1.2b | B0464.9 |  | O45605 | P35129 |  | H02I12.8 | M7.1 |
| Q95005 | Q19207 |  | C36B1.4 | F08F8.2 |  | O45605 | P50880 |  | H02I12.8 | F13B10.2a |
| Q19207 | Q22799 |  | F08F8.2 | T26A5.9 |  | O17915 | P46769 |  | K01G5.4 | B0393.1 |
| P39745 | O62305 |  | F43C1.2b | K11E8.1c |  | Q07750 | P10986 |  | C38C3.5b | M03F4.2a |
| P39745 | O16299 |  | F43C1.2b | F32D1.1 |  | Q95008 | Q95005 |  | F25H2.9 | C36B1.4 |
