# Supplementary material for: Targeting Protein-Protein Interactions for Parasite Control
Source: PLoS One. 2011 Apr 27;6(4):e18381. doi: 10.1371/journal.pone.0018381 (PMC3083401; doi:10.1371/journal.pone.0018381)
Supplement: Table S13 — Complete list of RNAi phenotypes sorted by bin. (DOC) [file pone.0018381.s021.doc]

| **RNAi**  **Pheno** | **Binned Group** |  | **RNAi**  **Pheno** | **Binned Group** |  | **RNAi pheno** | **Binned Group** |
| --- | --- | --- | --- | --- | --- | --- | --- |
| **Abs** | Embryonic_Lethal/Arrest |  | **Sck** | Growth_Defect |  | **Him** | Other |
| **Cpa** | Embryonic_Lethal/Arrest |  | **Cgr** | Growth_Defect |  | **Lin** | Other |
| **Cyk** | Embryonic_Lethal/Arrest |  | **Adl** | Larval_Adult_Lethal_Arrest |  | **Lpd** | Other |
| **Emb** | Embryonic_Lethal/Arrest |  | **Lva** | Larval_Adult_Lethal_Arrest |  | **Mec** | Other |
| **Etv** | Embryonic_Lethal/Arrest |  | **Lvl** | Larval_Adult_Lethal_Arrest |  | **Mut** | Other |
| **Led** | Embryonic_Lethal/Arrest |  | **Cll** | Larval_Adult_Lethal_Arrest |  | **Rde** | Other |
| **Let** | Embryonic_Lethal/Arrest |  | **Bli** | Morphology_Defect |  | **Fgc** | Sterility |
| **Mul** | Embryonic_Lethal/Arrest |  | **Bmd** | Morphology_Defect |  | **Glp** | Sterility |
| **Nmo** | Embryonic_Lethal/Arrest |  | **Clr** | Morphology_Defect |  | **Gon** | Sterility |
| **Npo** | Embryonic_Lethal/Arrest |  | **Dpy** | Morphology_Defect |  | **Lag** | Sterility |
| **Ocs** | Embryonic_Lethal/Arrest |  | **Lon** | Morphology_Defect |  | **Mei** | Sterility |
| **Oma** | Embryonic_Lethal/Arrest |  | **Pch** | Morphology_Defect |  | **Mel** | Sterility |
| **Oth** | Embryonic_Lethal/Arrest |  | **Rol** | Morphology_Defect |  | **Mig** | Sterility |
| **Pat** | Embryonic_Lethal/Arrest |  | **Rup** | Morphology_Defect |  | **Ooc** | Sterility |
| **Pna** | Embryonic_Lethal/Arrest |  | **Sma** | Morphology_Defect |  | **Ste** | Sterility |
| **Pnm** | Embryonic_Lethal/Arrest |  | **Cmo** | Morphology_Defect |  | **Stp** | Sterility |
| **Rot** | Embryonic_Lethal/Arrest |  | **Hya** | Movement_Defect |  | **Tum** | Sterility |
| **Sle** | Embryonic_Lethal/Arrest |  | **Prl** | Movement_Defect |  | **Cst** | Sterility |
| **Spd** | Embryonic_Lethal/Arrest |  | **Prz** | Movement_Defect |  | **Pro** | Unclassified |
| **Spn** | Embryonic_Lethal/Arrest |  | **Slu** | Movement_Defect |  | **Egl** | Vulval_Egg_Laying_Defect |
| **Spo** | Embryonic_Lethal/Arrest |  | **Unc** | Movement_Defect |  | **Evl** | Vulval_Egg_Laying_Defect |
| **Cel** | Embryonic_Lethal/Arrest |  | **Cmv** | Movement_Defect |  | **Muv** | Vulval_Egg_Laying_Defect |
| **Age** | Growth_Defect |  | **Ced** | Other |  | **Pvl** | Vulval_Egg_Laying_Defect |
| **Daf** | Growth_Defect |  | **Esp** | Other |  | **Pvu** | Vulval_Egg_Laying_Defect |
| **Gro** | Growth_Defect |  | **Fem** | Other |  | **Vul** | Vulval_Egg_Laying_Defect |
| **Mab** | Growth_Defect |  | **Fog** | Other |  | **Ceg** | Vulval_Egg_Laying_Defect |
| **Mlt** | Growth_Defect |  |  |  |  |  |  |
