# Supplementary material for: Targeting Protein-Protein Interactions for Parasite Control
Source: PLoS One. 2011 Apr 27;6(4):e18381. doi: 10.1371/journal.pone.0018381 (PMC3083401; doi:10.1371/journal.pone.0018381)
Supplement: Table S14 — Oligonucleotide probes used for FISH. Sense probes with the respective label were used as controls for each probe. (DOC) [file pone.0018381.s022.doc]

| Gene name | orientation | label | Sequence (5’-3’) |
| --- | --- | --- | --- |
| Q19126 [XP_00189449.1] | Anti-sense | digoxygenin | TCTAGGCAAGTGCTCTGTTTATTCA |
| O01427 [XP_001892118.1] | Anti-sense | biotin | TATTCAACGTAGCCTGACGAGT |
| P46822 [XP_001895440.1 ] | Anti-sense | digoxygenin | GTATCACCTCCTTTATGTTTATTCTCTTCACG |
| Q17581 [XP_001894559.1] | Anti-sense | biotin | TAT TCA ACG TAG CCG ACG AGT |
